# Ion-Doped Calcium Phosphate-Based Coatings with Antibacterial Properties

**DOI:** 10.3390/jfb14050250

**Published:** 2023-04-29

**Authors:** Marco Fosca, Alexandru Streza, Iulian V. Antoniac, Gianluca Vadalà, Julietta V. Rau

**Affiliations:** 1 Istituto di Struttura della Materia, Consiglio Nazionale delle Ricerche (ISM-CNR), Via del Fosso del Cavaliere 100, 00133 Rome, Italy; 2 Faculty of Material Science and Engineering, University Politehnica of Bucharest, 313 Splaiul Independentei Street, District 6, 060042 Bucharest, Romania; alexandru_streza@yahoo.com (A.S.); antoniac.iulian@gmail.com (I.V.A.); 3 Academy of Romanian Scientists, 54 Splaiul Independentei Street, District 5, 050094 Bucharest, Romania; 4 Laboratory of Regenerative Orthopaedics, Research Unit of Orthopaedic, Department of Medicine and Surgery, Università Campus Bio-Medico di Roma, Via Alvaro del Portillo 21, 00128 Rome, Italy; g.vadala@policlinicocampus.it; 5 Operative Research Unit of Orthopaedics, Fondazione Policlinico Universitario Campus Bio-Medico, Via Alvaro del Portillo 200, 00128 Rome, Italy

**Keywords:** antibacterial, coating, calcium phosphate, ion-doped, ion-substituted, bone implant

## Abstract

Ion-substituted calcium phosphate (CP) coatings have been extensively studied as promising materials for biomedical implants due to their ability to enhance biocompatibility, osteoconductivity, and bone formation. This systematic review aims to provide a comprehensive analysis of the current state of the art in ion-doped CP-based coatings for orthopaedic and dental implant applications. Specifically, this review evaluates the effects of ion addition on the physicochemical, mechanical, and biological properties of CP coatings. The review also identifies the contribution and additional effects (in a separate or a synergistic way) of different components used together with ion-doped CP for advanced composite coatings. In the final part, the effects of antibacterial coatings on specific bacteria strains are reported. The present review could be of interest to researchers, clinicians, and industry professionals involved in the development and application of CP coatings for orthopaedic and dental implants.

## 1. Introduction

Implant-associated infections present a significant problem in orthopedic and dental surgeries, affecting up to 5–10% of all implant interventions. These infections can lead to implant failure, long hospital permanence, and increased healthcare costs [1]. In addition, implant-associated infections can be life-threatening, particularly for immunocompromised patients or those with other co-morbidities [2]. One of the ways to address this problem is to develop implant coatings possessing antibacterial properties. Antibacterial coatings can help to reduce the risk of implant-associated infections by inhibiting the growth of bacteria on the implant surface [3,4]. Several different types of antibacterial coatings have been investigated, including metal coatings, polymer coatings, and ceramic coatings [3,5,6,7]. Calcium phosphate (CP) coatings have been developed for applications in orthopaedic and dental implants due to their biocompatibility, osseointegration, and their ability to induce bone formation [8,9]. To enhance the properties of CP coatings [10], substitution of calcium (Ca) ions with other ions, such as magnesium (Mg), strontium (Sr), zinc (Zn), etc., has been performed due to their beneficial effects on bone regeneration and mechanical properties [11,12,13,14,15,16,17,18]. Mg is known to enhance bone formation and remodeling [19], while Sr stimulates new bone formation and reduces bone resorption [20]. Zn has been shown to increase the mechanical strength and antibacterial properties of CP coatings [21]. Several studies investigated the effects of metal ions on the physicochemical, mechanical, and biological properties of CP coatings [22,23,24,25,26,27,28,29,30,31]. Due to their potential to impair bacterial biological processes, metal ions are utilized as antibacterial additions [10]. The three main mechanisms by which metal ions suppress bacteria are the following: enzymatic inhibition, oxidative stress, and membrane rupture [32,33]. In the first, metal ions hinder processes involving enzymes to fight against germ growth. Silver (Ag), copper (Cu), and Zn can decrease enzymes’ activity by binding to their active sites. Ag ions can be attached to enzymes’ thiol groups, inhibiting bacterial respiration and decreasing energy generation. Cu ions impede the DNA replication and protein synthesis of enzymes [34]. 

In the second mechanism, metal ions fight against microorganisms via oxidative stress, producing reactive oxygen species (ROS) that harm bacterial cells. ROS destroy proteins, lipids, and DNA, killing cells [35,36]. Cu ions produce hydroxyl radicals that harm bacterial cells [37,38], while Zn ions form ROS, which destroy bacteria [39]. 

The third antibacterial mechanism of action of metal ions is membrane rupture. Ag ions can induce the leak of bacterial membrane content, and Cu ions can damage bacterial membranes as well [38,40]. 

Metal ion antibacterial properties differ for various bacteria strains. Ag ions inhibit both Gram-positive and Gram-negative bacteria, but the mechanism of their action is different for these two groups. In Gram-positive bacteria’s peptidoglycan layer, Ag ions impede cell wall formation, while in Gram-negative bacteria they can damage the outer membrane [41]. Cu inhibits DNA replication in Gram-positive and Gram-negative bacteria. In particular, it was reported that Cu ions can potentially affect Gram-negative bacteria’s outer membrane [38]. Zn ions inhibit DNA replication and protein synthesis of enzymes of several bacteria strains. Zn ions form ROS, which destroy bacterial cells [42]. 

Moreover, metal ions have benefits beyond their antibacterial action. Ag has anti-inflammatory and wound-healing characteristics [43]. Cu ions increase angiogenesis, collagen production, and anti-inflammation processes [44,45,46]. Zn ions have anti-inflammatory, wound-healing, and collagen-synthesis characteristics [34]. Therefore, metal ions can be used to design new antibacterial materials with additional beneficial characteristics for tissues. 

A comprehensive understanding of the antibacterial mechanisms mentioned above, is an obligatory step for the development of a novel coating with effective antibacterial properties. Therefore, there is a strict correlation between a full comprehension of bactericidal mechanisms involving metal ions and their role in antibacterial coatings.

The objective of this systematic review is to critically evaluate the literature on ion-doped CP-based coatings for biomedical implant applications. Specifically, this review addresses the following key points:

1. The effects of ion addition on the physicochemical, mechanical, and biological properties of CP-based coatings.
2. The effects of additional components (not CP-based) in the most advanced composite coatings with ion-doped CPs as principal component.
3. The effects produced by antibacterial coatings on specific bacteria strains.

It should be noted here that the literature references search for this review was performed via the Web of Science platform by means of various queries, making use of different combinations of several keywords, such as hydroxyapatite, calcium phosphate, ion, coating, layer, film, deposit, substituted, doped, antibacterial, antimicrobial, and bacteria. These queries produced roughly 250 articles. Due to the fact that our rationale was to select only articles including antibacterial tests for ion-containing calcium phosphate coatings, this number was significantly reduced, and only approximately 35% of the articles were reviewed. It should be also added that only CP matrix as an ions container was considered in this review, due to its biomimetic properties. 

## 2. Ion-Doped Calcium Phosphate Coatings

### 2.1. Single Ion-Doped Calcium Phosphate Coatings

#### 2.1.1. Silver-Doped Calcium Phosphate (Ag-CP) Coatings

Inclusion of Ag within the calcium phosphate coating can induce modifications in its properties. Ag inclusion can take place both through the substitution of Ca ions by Ag ions in the CP lattice or via dispersion of metallic Ag particles within the CP matrix, which can agglomerate to form particles of different size. It should be taken into account that, in the most cases, Ag substitution into a CP lattice can occur together with its dispersion in the metallic form. Due to different factors, such as deposition parameters and Ag concentrations, one form of Ag inclusion can prevail over the others. Some works reported the presence of metallic Ag detected via X-ray diffraction (XRD) [47,48,49,50]. In all these papers, the presence of metallic Ag was detected only for a relatively high content of Ag, usually for concentrations equal to or higher than 3%. On the other hand, other studies reported a perfect integration of Ag into the host CP lattice, and no metallic Ag was detected [51,52,53,54,55,56]. 

Interestingly, in some works [56,57,58,59], when metallic Ag was introduced in concentration equal to or lower than 0.002 mol%, it was below the limit of the XRD technique, and no detection of the metallic Ag phase was registered. In contrast, the XPS method, even despite the small amount of Ag present in the hydroxyapatite (HA) matrix, was able to detect Ag in different oxidation states, revealing the presence of a small amount of Ag^0^ (metallic) (ranging from 2–13% of 100%) along with Ag^+^ (23–27%) and Ag^2+^ (60–75%). The study [59] used two different deposition approaches, which allowed the authors to obtain coatings containing Ag either (1) as micro-sized Ag phosphate particles embedded in the CP matrix (Ag-CP(1)), or (2) as metallic Ag nanoparticles on the CP coating (Ag-CP(2)). The XRD pattern of the Ag-CP(2) coating showed peaks belonging to pure metallic Ag. In the Ag-CP(1) coating, no diffraction peaks related to metallic Ag were observed, but well-distinguished diffraction peaks of Ag_3_PO_4_ were detected, even though XPS also revealed the presence of Ag^0^ (12%). 

Ag can influence the crystallinity of the host CP matrix, either through dispersion or substitution of Ca^2+^ ions in the CP lattices. Tunneling electron microscopy (TEM) characterization of Ag dispersed in fluoro-hydroxyapatite (FHA) revealed the presence of a crystallinity gradient in the investigated coatings, passing from the crystalline structure of the bottom layer to the mainly amorphous one of the top layer [60]. This arrangement was attributed to the sputtering deposition conditions and, in particular, to the substrate temperature affecting the crystallinity of the deposited material [61,62]. Moreover, Ag particles were homogenously distributed throughout the entire coatings, with typical dimensions ranging from 5–30 nm, and up to 50 nm in the case of higher Ag content. In [56], Raman spectroscopy investigations revealed that the incorporation of Ag ions into the apatite structure caused shifts in the peak positions, reflecting a decrease in coating’s crystallinity.

The substitution of Ca ions by Ag ions into the CP (mainly HA) lattice also affects lattice parameters, interplanar distances and, more generally, crystallites size. Various literature references [50,55,58] agreed on an enlargement of the above mentioned parameters due to the inclusion of Ag ions accommodated in the Ca^2+^ lattice sites. The higher ionic radius of Ag^+^ (0.128 nm) compared to that of Ca^2+^ (0.099 nm) can explain the variations in the crystal lattice parameters. Greater parameter sizes, including grain size of crystal domain, were found in concomitance to the higher level of Ag substitution.

Surface morphology was investigated mainly by means of scanning electron microscopy (SEM) and atomic force microscopy (AFM) techniques. Nano-sized superficial aggregates and a needle-like morphology represented the most common situation for Ag-CP coatings. The deposited films homogeneously covered the substrate surface with quite smooth layers and no visible cracks when deposited via pulsed laser deposition, physical vapor deposition, plasma electrolytic processing, electrophoretic deposition, and radio frequency (RF) sputtering deposition methods [48,49,52,53,59,60]. It is worth noting that the surface morphology evolved depending on deposition time. Small spherical droplets characterized films deposited over short deposition times. As the time increased, the droplets underwent coalescence and aggregated in larger islands, and, finally, formed a homogeneous layer that completely covered the substrate surface. Ag substitution was also found to influence coating morphology. It induced the formation of fine needles, elongated and stacked along the main axis. The increase in the Ag content led to a more sharpened morphology of crystallites: morphing from 90 nm in diameter and 400 nm in length to 50 nm in diameter and 300 nm in length [56].

Roughness of coatings was found to diminish depending on the Ag content and deposition time. Root mean square (RMS) values of 22, 17, and 14 nm were found for HA coatings with Ag concentrations of 1%, 3%, and 5%, respectively [50]. In another study, the dependence of roughness on deposition time was reported. Values of RMS of 52, 69, and 78 nm were measured for deposition times of 10, 20, and 30 min, respectively [55]. These results are coherent with other structural analysis outcomes, confirming that the presence of Ag leads to the formation of smaller particles and, consequently, a smoother surface.

The hydrophilicity of Ag-CP coatings was investigated by measuring their wet contact angle. Authors [63] reported that the observed contact angles for both HA and Ag-HA were lower compared to those of a Titanium (Ti) substrate. In another study [51], it was demonstrated that Ag-HA surface was characterized by a significantly lower value of wet contact angle, compared to pure HA. A higher hydrophobicity of coatings is an important feature that helps to prevent early stages of bacteria adhesion.

It should be noted that wettability is an important characteristic of biomaterials, since it influences not only bacteria adhesion, but also protein attachment and cell adhesion and proliferation. The ideal water contact angle for biomaterial surfaces is considered to be in the range of 35–80°; accordingly, the material should have a moderately hydrophilic surface [64].

Ag-CP coatings’ adhesion to a Ti substrate was investigated using the adhesion standard test method (ASTM) D3359-09 for the as-deposited films obtained by sputtering deposition [60]. The evaluation of coatings after their immersion in the phosphate-buffered saline (PBS) was investigated via SEM-energy dispersive X-ray spectroscopy (EDS). The obtained results, in both cases, allowed the inference that the interface between the Ag-CP coatings and Ti substrate can endure mild to moderate destabilizing forces [60]. In another study [54], scratch tests were conducted on Ag-HA coatings deposited via electrostatic spraying, which underwent heat treatment at different temperatures: 500, 700, and 900 °C. Good adhesion strength with no evident cohesive failure was found for coatings treated at high temperatures (700 and 900 °C). This behavior was attributed to a mutual diffusion of ionic species between the substrate (Ti) and coating (Ag-HA), and the consequent creation of a stable mechanical interface. After 4 weeks of immersion in PBS, the coatings were partially detached, and a large area of the Ti substrate was exposed to the solution. Moreover, EDS quantitative analysis highlighted a significate loss of Ag in the residual film.

A corrosion assay was conducted to assess the resistance of coatings in physiological solutions. The potentiodynamic polarization technique was used as an appropriate tool to test coatings’ resistance against possible delamination phenomena induced by in vitro conditions. A significant improvement in corrosion behavior was found for Ag-CP coatings in an experiment performed in both physiological (pH 7.4) and slightly acidic (pH 4.5) solutions [52,58]. In [50], it was reported that, by increasing the Ag content (1%, 3% and 5%), the values of current density (I_corr_) were decreased, and corrosion potential (E_corr_) was simultaneously increased. The shift of E_corr_ to higher values, registered in correspondence with the higher Ag concentration, testified to a better corrosion protection compared to the Ti surface. In [56], electrochemical impedance spectroscopy (EIS) showed a higher conductivity and a slight decrease in corrosion resistance upon the addition of Ag to FHA. This behavior was correlated with the influence of Ag present in a chemical solution micro-environment, characterized by a higher conductivity.

Ag release investigations from Ag-CP coatings are of particular importance, allowing the detection of the quantity of Ag present in the release medium at different time-points. The concentration of Ag in solutions is directly related to its antibacterial potential. Various works reported a long-term release of Ag over the course of 7 to 10 days [47,53,57,58,60]. For a relatively low Ag concentration in CP (up to 1.5%), 0th order release is generally observed, whereas, for a higher Ag concentration, it is 1st order release and a typical diffusion profile that represents the most common Ag dissolution behavior. After an initial fast release, a decrease in its rate is generally observed after 7 days. The release rate depends on several factors; among them, the most important are the amount of Ag in the coating and the degree of crystallinity of the deposited coating: the crystalline phase is characterized by a slower Ag release, while the amorphous phase usually displays a faster Ag release. Almost all the analyzed literature confirms that the minimum inhibitory Ag concentration (0.1 ppm) necessary to achieve an effective antibacterial performance is reached after a few hours from the beginning of the release. 

The Ag release profile is also affected by the oxidation state: Ag^+^ showed a burst release, while metallic Ag particles were characterized by a slower and sustained elution and a higher cumulative concentration [59]. Finally, deposited films with an initial Ag content of 2–3% achieved Ag levels of approximately 1.6 ppm in the release solution, this value being below the cytotoxicity level [47,57,58,65,66,67].

The biocompatibility of coatings is one of their most important characteristics, since minimal or no cytotoxicity is required for living cells and tissues. In [59], the biocompatibility tests were conducted using osteosarcoma cells. An Ag-CP coating characterized by the presence of Ag^+^ was found to be cytotoxic towards the cells, showing almost zero alive cells on the coating. On the other hand, the Ag-CP coating with metallic Ag exhibited better biocompatibility, presenting an unaffected viability and morphology of the cells [59].

#### 2.1.2. Zinc-Doped Calcium Phosphate (Zn-CP) Coatings

Successful inclusion of Zn within the CP structure was reported by many authors [68,69,70,71,72]. An effective substitution of Ca^2+^ by Zn^2+^ within the CP lattice was attested by XRD studies reporting slight modifications of lattice parameters due the difference between the ionic radii of Zn^2+^ (0.074 nm) and Ca^2+^ ions (0.099 nm) [69,70,71,72].

No evidence of metallic Zn presence within the coatings was reported in the investigated literature [68,69,70,71,72], and it can therefore be generally asserted that Zn^2+^ ions can be easily introduced into the CP lattice. Nevertheless, a slight increase in the Ca/P ratio (2.2–2.3) was observed [73].

Zn-substituted CP coatings were characterized by smaller particle sizes, compared to the unsubstituted counterparts, with typically sub-micrometric dimensions, usually in a range below 100 nm, as attested by XRD, TEM, and SEM measurements [68,69,70].

The morphology of Zn-CP coatings can be influenced by different factors, such as deposition method and conditions, and concentration of Zn. A needle-like morphology was reported for Zn-CP coatings deposited via electrophoretic deposition, with initial Zn content ranging from 0 up to 10 at% [32]. Other literature references report particles of a spherical shape [69] and, more specifically, a transition from spherically shaped particles (lower Zn content) to a hexagonal rod-like structures (higher Zn content) [70,72]. The surface morphology of the obtained coatings mainly appeared as smooth, homogeneous, and crack-free. On the other hand, surface roughness was sensitive to several factors, such as Zn content and deposition parameters. Zn-CP-based coatings deposited via the electrophoretic method with various Zn concentrations showed RMS ranging from 2.6 nm for the unsubstituted CP coating (0% Zn) up to 6 nm for coatings with 10 at% of Zn [68]. In another paper [72], a Zn-CP film prepared using an electrochemical deposition method with a roughness of 640 nm was reported. Vertical cracks and micropores characterized the inner morphology of the Zn-CP coating prepared via the electrospray method [69]. In [73], no significant morphology modification was observed for a Zn-CP coating (with low concentration of Zn (0.4–0.8 wt%)) prepared via magnetron sputtering and characterized by a roughness of 0.23 ± 0.06 μm.

Only a few data on the wettability of Zn-CP coatings are available in the literature. In a recent work [72], a contact angle of 62° for an electrochemically deposited Zn-CP coating was reported. This value is high enough to both favor bioactivity and foster bacteria adhesion. 

The mechanical properties were also reported in [72]. The authors found that, for the Zn-CP film, the adhesion strength was in the range of 8–9.5 MPa. In [73], the adhesion of Zn-CP coatings deposited on different Ti alloys (Ti-6Al-7Nb and Ti–6Al–4V) was investigated through the scratch test, revealing that the coating adhesion can be significantly influenced by the substrate. Critical load failure values of 27.8 N and 15.5 N were registered for Zn-CP deposited on Ti-6Al-7Nb and Ti–6Al–4V, respectively.

Zn-substituted CP coatings were characterized by an enhanced corrosion resistance with respect to the unsubstituted ones in a simulated body fluid (SBF) environment [69]. As reported in [70], E_corr_ was measured for both Zn-CP and CP coatings, and values of −367 mV and −278 mV, respectively, were registered. Therefore, the inclusion of Zn ions within the CP lattice induced a shift of E_corr_ towards more negative values. The enhanced corrosion resistance implied an inhibition of the Zn ions’ diffusion from the sample.

The stability of nanocrystalline Zn-CP coatings within a Dulbecco’s modified eagle medium (DMEM) solution was investigated in [68]. It was proven that the films remained almost unchanged during the first 3 days of immersion, while afterwards they started to gradually dissolve, resulting in a smooth surface. 

The release of Zn^2+^ and Ca^2+^ ions was investigated in [69]. The authors reported two different release profiles for different Zn concentrations observed during a period of 14 days. In the coating with 5 mol% of Zn, the dissolution in SBF was registered during the first 7 days, followed by a decrease for the remaining 7 days, whereas a continuous release from day 1 to day 14 characterized the behavior of the CP coating substituted with 10 mol% of Zn. Biodegradation of coatings and release of Zn and Ca ions can be also influenced by the substrate, as demonstrated in [73], where Zn-CP coatings on Ti–6Al–7Nb showed a twofold increase in the dissolution rate, compared to Zn-CP on Ti–6Al–4V.

The bioactivity assay of a Zn-CP film was carried out in SBF solution in [69]. The formation of a hydroxy-carbonate apatite (HCA) layer after 14 days of immersion was attested by spectroscopic (Raman) and morphological (SEM) characterizations. CO_3_^2−^ Raman bands confirmed the presence of carbonated apatite, while the characteristic porous cauliflower-like texture with the presence of cracks characterized the morphology of the coating layer. By comparing coatings with different Zn contents, the authors concluded that Zn could inhibit the formation of the precipitated apatite film, thus leading to a decrease in bioactivity.

The cytotoxicity test revealed that Zn-CP coatings may induce different effects, depending on cell type. It was observed that the Zn-CP coatings did not significantly affect the differentiation of human amniotic mesenchymal stem cells (hAMSC) into osteoblasts. On the other hand, experiments conducted on tumoral Jurkat T cells showed an increased number of dead cells and reduced viability after 48 h of culture [73].

#### 2.1.3. Copper-Doped Calcium Phosphate (Cu-CP) Coatings

Coatings based on Cu-CP materials with antibacterial properties have been investigated and reported in [74,75,76,77,78]. Insertions of nanoCu [74,75] in the CP deposited films, according to the cited authors, resulted in the substitution of Ca^2+^ by Cu^2+^ ions within the CP lattice; this conclusion was mainly made based on the absence of metallic Cu in the XRD spectra. Authors [74,77] investigated the introduction of Cu ions from a Cu nitrate precursor in the CP deposited films, and proved the substitution of Ca ions by Cu ions based on the shifts of the diffraction peaks and lattice parameters variation. The reduction in lattice parameters, shrinkage of the unit cell, and distortion of the hexagonal symmetry of CP were observed and associated with the accommodation of Cu^2+^ ions (ionic radius of 0.073 nm) in the Ca^2+^ lattice site (Ca^2+^ ions are characterized by a larger ionic radius (0.099 nm) [74]. Additionally, the formation of new phases, such as beta tricalcium phosphate (β-TCP) and calcium pyrophosphate, was also registered [78]. Further confirmation of Cu insertion in the CP lattice was provided by the comparison of the Fourier transform infrared (FTIR) spectra of Cu-substituted and unsubstituted CP coatings, in which a shift in the intensity variations of the PO_4_^3−^ band was registered [77]. Electron paramagnetic resonance spectroscopy (EPR) investigations also confirmed the inclusion of Cu^2+^ ions into the TCP structure within a distorted rhombic configuration [78]. Along with this, some works attested the presence of metallic Cu or CuO in the investigated coatings [76,77]. Other references reported the presence of Cu in an oxidized state [74,75]. 

The morphology investigations reported that the presence of Cu^2+^ ions in the coating solution (the coatings prepared by electrochemical deposition) restrained the precipitation, resulting in a slight decrease in crystal size [74]. Cu substitution in the HA coatings increased their density and reduced their porosity compared to the HA coatings without copper. Both the HA and Cu-HA coatings were characterized by a smooth surface and thickness of approximately 10 μm. The EDS elemental mapping showed a uniform distribution of both Cu and Ca across the entire surface [74]. Cu-TCP ceramic samples obtained by mechano-chemical activation synthesis showed an increased crystallite size and porosity upon the addition of Cu [78]. Furthermore, the addition of Cu nanoparticles to the coatings reduced the number of cracks as a result of diminished internal stresses upon thermal processing. In [76], nanoHA coatings containing nanoCu were characterized by a lower thickness and enhanced nanomechanical properties, compared to nanoHA coatings without copper. For nanoHA coatings with Cu nanoparticles, an increase in roughness with the rise in nanoparticles content in the suspension was observed, but only for smaller metal nanoparticles in the electrolyte. In the coatings containing larger Cu nanoparticles, a reverse effect was revealed in [76]. In [77], the incorporation of Cu into the HA/functionalized–multi wall carbon nanotube (MWCNT) composite film resulted in an octahedral structure, which was different compared to the morphology of HA/MWCNT without Cu. The TEM analysis also confirmed the formation of octahedral crystals on a stainless steel surface. Particle agglomeration was reduced after the Cu incorporation, leading to an increased surface area, thus making it suitable for antibacterial and corrosion resistant applications [77]. 

Mechanical tests conducted in [76] reported that nanoHA coatings with an average particle size of nanoCu of 40 nm showed the highest values of nano-hardness and Young’s modulus, due to the increased homogeneity of the coatings and the reinforcing effect of nanoCu. The addition of metallic nanoparticles to the coatings increased the adhesion of the coatings to the Ti13Zr13Nb substrate. Among the nanoHA with nanoCu coatings, the highest adhesion was registered for the nanoHA with average nanoCu particle size of 80 nm [76]. The coatings with nanoCu particles possessed an average contact angle lower than the Ti13Zr13Nb substrate due to their porous structure, while the nanoHA/nanoCu’80 coating was characterized by the highest contact angle among the coatings due to the high concentration of relatively large nanoparticles [76]. 

Thermal analysis conducted on Cu-TCP powder showed that Cu incorporation (0.1 wt%) into TCP increased its thermal stability. The lack of polymorphous transformation of β- to α-TCP in 0.1Cu-TCP at high temperatures indicated the stability of β-modification due to the substitution of Ca^2+^ by Cu^2+^ [78].

Solubility is also influenced by Cu substitution. Comparing Cu-TCP and un-substituted TCP, a higher solubility of the former in a buffered TRIS solution was observed. This behavior was attributed to the substitution of Cu^2+^, which induced a crystal lattice distortion, reduced bond enthalpy and facilitated hydrolysis, thus leading to the increase in the solubility of Cu-TCP [78].

Corrosion assessment carried out via the potentiodynamic polarization test revealed that the Cu-HA coating was characterized by better barrier characteristics for the Ti substrates compared to other coatings without Cu, thus providing a superior corrosion protection in an SBF environment [74]. Indeed, the Cu-HA coating had a decreased specific surface and grain size, with respect to the HA coating, resulting in elevated electron activity at grain boundaries, decreased I_corr_, and, thus, an improved corrosion protection. In [77], the Cu-substituted HA/f-MWCNT composite coating provided higher barrier properties for a stainless steel substrate. In particular, a remarkable decrease from 6.8 to 3.8 μA for the corrosion I_corr_ was registered, suggesting that the presence of Cu improved the coating’s protective ability against the corrosive environment of body fluids, such as sweat and saliva [77]. 

The bioactivity of biomaterials was studied by observing the precipitation behavior of bone-like apatites in SBF [74,77]. The surface morphology and structural features of the HA and Cu-HA coatings after their soaking in SBF were investigated by SEM and XRD [74]. The results indicated that the Cu-HA coating induced apatite nucleation and growth on the Ti surface after soaking. 

Finally, the cytotoxicity features of Cu-HA were also investigated in [74,78]. SEM images revealed that MC3T3-E1 osteoblast-like cells were well spread onto all the samples and exhibited good viability on both the Cu-HA and HA films, indicating that Cu^2+^ ions do not affect the cell morphology. The obtained Cu-HA coating (with 0.80 wt% Cu^2+^) showed good cytocompatibility towards osteoblast cells [74]. In [77], the DMEM medium containing 5 wt% of the Cu-HA/f-MWCNT sample was used to assay human osteoblast cells’ viability. The obtained MTT results provided evidence that, in the sample without Cu, more than 97% of viable cells were observed, while in the sample with Cu this was approximately 95%, thus allowing the authors to conclude that the investigated Cu-HA/f-MWCNT sample was non-cytotoxic and biocompatible. Good cytocompatibility of a Cu-TCP sample (0.1 wt% of Cu) was also observed in [78], where the MTT test showed 100% metabolic activity for mouse fibroblast NCTC L929 cells and the Resazurin test displayed viability near to 100% for dental pulp stem cells.

#### 2.1.4. Magnesium-Doped Calcium Phosphate (Mg-CP) Coatings

The incorporation of Mg^2+^ ions within HA/MWCNT coatings caused a broadening of and reduction in the HA peak intensity, resulting in a decrease in the HA lattice parameters and an increase in the lattice strain due to the smaller ionic radius of Mg^2+^ (0.069 nm), compared to Ca^2+^ (0.099 nm); the coatings’ crystallite size decreased from 50 nm to 40 nm [79]. After the addition of Mg into HA, the peak associated with HA(002) shifted towards higher angles, indicating that Mg^2+^ ions replaced Ca^2+^ in the HA lattice, according to [79,80]. The authors also reported a shift in the PO_4_^3−^ stretching vibration at 1127 cm^−1^, which could refer to the distortion occurring in the HA lattice upon the Mg substitution [79]. 

The morphology of the Mg-HA/MWCNT coating revealed spherical-shaped apatite crystals with an interconnected network-like microstructure after the Mg substitution [79]. It was also observed that the addition of different concentrations of Mg to the HA lattice resulted in a change from elongated ribbon-like crystals to wider ones. In [80], the coating roughness was measured using stylus profilometry (a mechanical probe-based method able to replicate surface profile), and the results showed that the average roughness increased with the Mg addition: an 8-times increase was registered when the Mg quantity was increased from 1 to 1.5 mM. Moreover, the presence of Mg^2+^ ions in the electrolyte inhibited the HA crystallization, resulting in a porous, irregular, and heterogenous coating [80]. 

Adhesion assay results showed that the best adhesion to the substrate (a Ti alloy) was detected for coatings with an Mg concentration of 1 mM [80]. Indeed, further increase in the Mg amount caused delamination/detachment along the cutting line and inside the squares of samples prepared for testing according to the ASTM D3359-17 [81].

Potentiodynamic polarization measurements demonstrated an increased corrosion resistance of the Mg-HA/MWCNT due to the lower I_corr_ value, when compared to the HA/MWCNT-coated stainless steel [79], whereas in [80], on the contrary, the E_corr_ and I_corr_ values obtained for the Mg-HA coatings suggested that the solubility of HA increased with the addition of Mg. The polarization resistance values were the highest for undoped HA coatings, followed by those with 1 and 1.5 mM of Mg, allowing to conclude that a higher dissolution rate is reached for the coatings with an increased Mg content [80].

### 2.2. Multiple Ion-Doped Calcium Phosphate Coatings

Preparation of Cu,Ag-doped HA coatings was realized via the ultrasonic coupled sol–gel technique and reported in [82]. Samples were labeled according to the molar ratio of Ag and Cu, i.e., Cu1.0 (100% Cu, 0% Ag) and Ag1.0 (0% Cu and 100% Ag). Lattice parameters of the samples were close to those of the stoichiometric HA600 (HA calcinated at 600 °C), indicating, according to the authors [82], a small amount of Cu^2+^ and Ag^+^ ions substitution in the crystal lattice. The crystallite size of HA600 was larger than that of Cu1.0-doped HA600 and smaller than that of Ag1.0-doped HA600, which was attributed to the differences in ionic radii (their values are mentioned in the text above). Additionally, in this work [82], the presence of Ag^+^ and Cu^2+^ ions in the HA structure caused lattice parameter and crystallite size modifications, as confirmed by XRD investigations.

Ag and silicon (Si) were incorporated into a HA matrix to prepare an Ag-doped HA/calcium silicate (CS) (Ag-HA/CS) layer deposited on Ti modified with TiO_2_ nanotubes (TN) [83]. Authors reported a slight increase in the unit cell parameters accounting for the Ag^+^ ions’ incorporation in the Ca^2+^ sites of the HA lattice, while various IR bands related to SiO, Si-O-Si, SiO_3_^2−^, Si-O-Ca bending modes testified the inclusion of Si. The Si^4+^ ions considerably boosted the adhesion strength of the coating to the substrate due to ceramic particles entering the spaces in TN and providing mechanical interlocking. Additionally, the thermal expansion coefficient of CS, close to that of Ti and its alloys, allowed a favorable interface bonding between the substrate and coating, compared to traditional HA coatings. In the coatings containing both the ions, potentiodynamic polarization tests demonstrated an increase in the corrosion resistance with respect to the undoped coatings. With respect to the uncoated samples, the Ag-HA/CS layer deposited on Ti modified with TN demonstrated an increased wettability (contact angle of 0° for the coating, 53.6° for Ti, 141° for Ti modified with TN) and an enhanced apatite forming ability, attested upon bioactivity investigation. The cytotoxicity assays revealed a better MC3T3-E1 osteoblast cell viability when coated with HA or Ag-HA/CS (HA or Ag-HA/CS giving similar results) than when left uncoated, and the cell differentiation capacities were also significantly enhanced.

Si and Ag were used to prepare coatings composed of nano-sized Si-HA:Ag-HA = 1:3 (1Si-HA-3Ag-HA) on a glass substrate using the drop-on-demand micro dispensing technique in [84]. The biocompatibility was demonstrated for human adipose-derived mesenchymal stem cells (hASCs), studying their growth on the HA, Si-HA, Ag-HA, and 1Si-HA-3Ag-HA coatings. A significant increase in the hASCs’ proliferation on the coatings was registered for the time period of the first 4 days; after that, from day 4 to day 7, cell differentiation was promoted on the Si-HA and 1Si-HA-3Ag-HA coatings; and afterwards, from day 7 to day 10, cell proliferation increased again along with their differentiation for all the coatings. According to the authors [84], this fact could be linked to the regulation of cellular responses by the Si^4+^ ions. Moreover, the solubility of the Si-HA coatings was improved due to the reduced particle size of Si-HA, which prompted a fast ion (Ca^2+^, P^5+^, and Si^4+^) release to the surrounding environment, promoting cell proliferation and differentiation. The results of this investigation also demonstrated that the integration of Ag had no discernible influence on cell proliferation, indicating that the Ag-HA (1.5 wt% Ag) coatings did not induce noticeable cytotoxic effect. 

Sr and Ag were incorporated into a HA coating deposited on a Ti surface via the hydrothermal method reported in [85]. The XRD patterns of the prepared coatings revealed that HA was the primary component, and the peak shifts versus lower 2theta values accounted for a partial substitution of Ca by Sr and Ag into the HA lattice. Inductively coupled plasma emission spectroscopy (ICP) provided information on the relative amounts of Ca, P, Sr, and Ag. SEM investigations testified to a uniform distribution of Sr,Ag-HA nanoparticles on the Ti surface, with the average size being less than 100 nm. A much-reduced water contact angle (30.8°) was observed on the coatings compared to pure Ti (57.5°), indicating an increase in hydrophilicity. The AFM and TEM images revealed that the addition of Sr led to an increase in the surface roughness, from 11 nm for the Ti surface to 550 nm for the HA coating and up to 809 nm for the Sr,Ag-HA coating. The Sr,Ag-HA coating exhibited long-term, sustained Ag^+^ release, indicating that the addition of Sr to the Ag-containing coating did not have a significant effect on the coating’s degradation kinetics. If compared to pure Ti, the proliferation activity of MG63 osteoblast-like cells on the Sr,Ag (Ca_8·95_SrAg_0_._1_(PO_4_)_6_(OH)_2_) coatings was much better than that on the Ag0.1 (Ca_9·95_Ag_0_._1_(PO_4_)_6_(OH)_2_) coatings. The alkaline phosphatase (ALP) activity results further indicated that the MG63 cells’ ALP activity on the Sr,Ag-HA coatings was much higher than that on the Ag0.1HA coatings.

Sr and Ag were used as co-doping ions for the fabrication of HA/TN bilayer coatings via the electrodeposition method [86]. The Sr,Ag-HA possessed increased lattice parameter characteristics compared to the stoichiometric HA. Due to the larger ionic radii of Sr (0.118 nm) and Ag (0.126 nm) compared to that of Ca (0.099 nm), the substitution of Ca^2+^ by Sr^2+^ and Ag^+^ in the HA crystal lattice was responsible for the rise in lattice parameters (from 114–116 pm to 132 pm). On the nanotube framework, a homogenous, compact, and crack-free Sr,Ag-HA coating was deposited with an estimated thickness of 25 μm. An increased bond strength was a result of Sr,Ag-HA/TN anchoring, enhanced adhesion at the Sr,Ag-HA/anodized alloy interface, and mechanical interlocking offering a suitable scaffolding. The inclusion of TN as an intermediary oxide layer boosted the interface cohesive force by enhancing the chemical bond between Ti and the coating. Both the HA and Sr,Ag-HA surfaces exhibited good hydrophilicity, with contact angles of 7.1° and 25.6°, respectively. Compared to the pure Ti surface, such modified coatings displayed a superior resistance to corrosion in an SBF environment. Bioactivity studies in the SBF solution demonstrated an increase in apatite formation for the Sr,Ag-HA coatings.

Ag and Sr single-substituted and co-substituted HA powders were prepared by means of wet precipitation from cuttlefish bone with different amount of substitution (0, 1, 2.5, and 5 mol%) for each ion species in [87]. XRD patterns of the investigated powders showed sharp peaks of HA and β-TCP phases, indicating their crystalline state, with no additional peaks of Ag and Sr. The lattice parameters of the HA phase increased linearly with the increase in the amount of Sr^2+^ ions substituted, while an irregular increase was observed by incrementing the Ag^+^ ion content. FTIR investigation revealed the shift of bands related to the PO_4_^3−^ group to lower wavenumbers, along with the attenuation of the OH^-^ band intensity. These results suggested that the Ag^+^ and Sr^2+^ substitutions in HA led to structural disorder along the c axis, increasing the amorphous phase content. SEM investigation revealed that the Ag^+^ and Sr^2+^ ion substitutions resulted in morphological modifications from a flower-shaped arrangement of crystals, characteristic of pure HA, to the smaller irregular crystal texture of substituted HA. The micrographs showed no significant difference in morphology between the powders of HA substituted with 1, 2.5, and 5 mol% of Ag and Sr; EDS mapping confirmed the uniform distribution of Ca, P, Ag, and Sr, but the amount of Ag was lower than expected with regard to precursor concentration. Adsorption of the bovine serum albumin (BSA) protein onto the non-substituted HA and Ag,Sr-HA powders was conducted under simulated physiological conditions (pH = 7.4). The Ag,Sr-HA powders showed higher affinity for BSA adsorption than the non-substituted HA due to more positive surface charges and larger surface areas. Zeta-potential (ZP) was determined in a PBS solution at different pH values. All the Ag,Sr-HA powders had a negative charge, and the ZP values decreased as the buffer pH increased. Additionally, the ZP became more negative as the degree of Ag and Sr substitution increased. It was also observed that the extent of BSA protein adsorption was relatively independent of the content of Ag and Sr, suggesting that the surface adsorption sites were saturated with no regard to substitution degree. Cytocompatibility evaluation was assessed via MTT test, performed on human embryonic kidney cells (HEK 293) and human mesenchymal stem cells (hMSC) by culturing the cells with the extract of each powder. After 3 days of the HEK 293 cell culture, the Ag,Sr-HA (2.5%) and Ag,Sr-HA (5%) powders showed a significant decrease in the cells’ viability compared to the non-substituted HA, while no significant difference was observed for the other samples, indicating a mild cytotoxicity of the former. The MTT test conducted on hMSCs showed no significant difference in the cell viability after 1 day of culture. However, after 3 days, a significant increase in cell viability for the Sr-HA (2.5%), Ag-HA (2.5%), and Ag,Sr-HA (1%) powders was registered. These findings suggest that various cell types showed different responses according to the powders’ characteristics.

**Figure 1:**
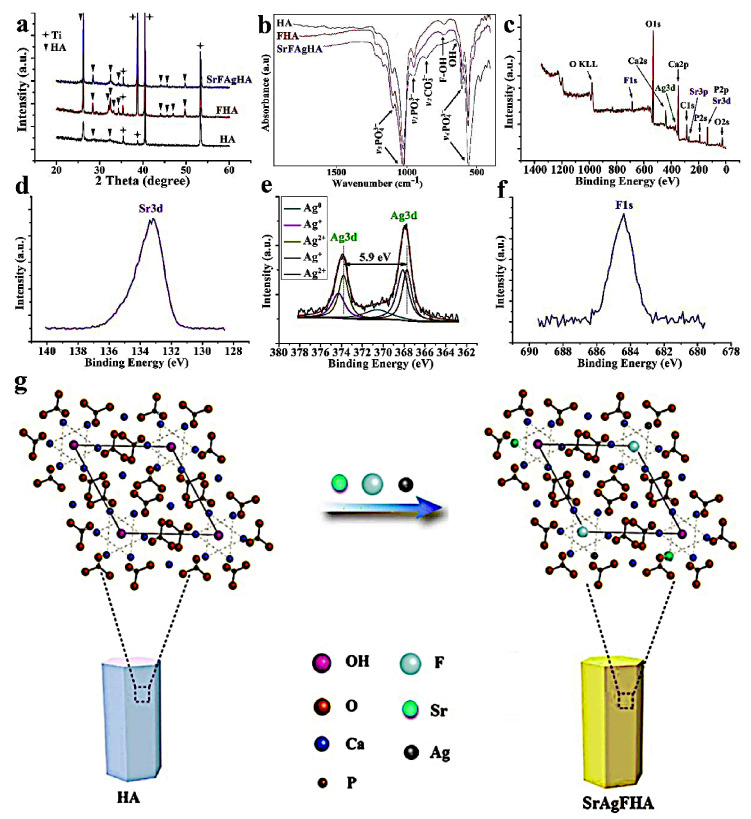
(**a**) XRD spectra of HA, FHA and Sr,Ag −FHA; (**b**) FTIR spectra of HA, FHA and Sr,Ag −FHA; XPS collected for Sr,Ag −FHA (**c**), high −resolution spectra of Sr 3d (**d**), Ag 3d (**e**), F 1s (**f**). (**g**) Possible molecular simulation of Sr,Ag −FHA structure. Reprinted with modifications from “Ceramic International 46, Zhang, X. et al., Chemical stability, antibacterial and osteogenic activities study of strontium-silver co-substituted fluorohydroxyapatite nanopillars: A potential multifunctional biological coating, 27758−27773, 2020” with permission from Elsevier.

A representation of the effect of addition of Ag and Sr ions into HA matrix is reported in Figure 1.

Ag and Zn-modified HA coatings were prepared by electrochemical deposition and investigated in [88]. Both Ag and Zn affected the plate-like morphology of HA. Agglomerations of Ag particles was observed in the Ag-HA coatings. The Zn-HA coatings were characterized by an interconnected network made of very thin and small crystals, suggesting that the morphology of HA was modified upon the addition of Zn. The bioactivity studies revealed that the growth rate of a new apatite layer on the Ag-HA, measured after 1, 3, 7, 14, and 21 days, was the fastest at each time interval, followed by the Zn-HA and HA coatings. Staining of human embryonic kidney (HEK 293T) cells for cytoskeletal proteins, such as actin and tubulin, indicated a reduced polymerization of actin in HA, Zn-HA, and Ag-HA coatings, compared to Ti samples, indicating that the HA coating effected the filamentous organization of actin. The cells grown on the Zn-HA and Ag-HA coatings had a higher proliferation rate, suggesting that employing these coatings to improve cell growth parameters may be advantageous. All coatings induced an increase in cell proliferation with respect to pure Ti; Ag-HA and Zn-HA coatings exhibited the best results. The HEK 293T cell viability findings demonstrated that all the coatings were cytocompatible, while the best results were achieved for the Zn-HA coatings.

Sr and Cu were incorporated into a co-substituted HA based coatings via the electrodeposition technique [89]. The presence of Sr^2+^ and Cu^2+^ led to a decrease in the coating’s roughness and to the formation of smaller nuclei, with respect to the unsubstituted HA. The ion release analysis performed via inductively coupled plasma mass spectrometry revealed a sustained release rate of Sr and Cu ions within 2 weeks. Electrochemical corrosion experiments revealed a higher corrosion resistance with a lower penetration rate for the Sr,Cu-HA coating with respect to the uncoated Ti. It was also shown that the HA and Sr,Cu-HA coatings were characterized by a higher adhesion strength to the Ti substrate. Finally, an increase in osteoblast adhesion, proliferation, and alkaline phosphatase activity was registered.

Co-substituted Sr,Zn-HA and pure HA coatings at different concentrations of Sr and Zn (5 mol% Sr,10 mol% Zn-HA, 10 mol% Sr,5 mol% Zn-HA, and 10 mol% Sr,10 mol% Zn-HA) were deposited via the plasma spray technique on a Ti alloy surface [90]. XRD analysis indicated that co-substitution of HA with Zn and Sr inhibited the formation of the HA structure and promoted the appearance of admixture phases, such as α-TCP, calcium-zinc-phosphate, and CaO. The presence of Sr^2+^ and Zn^2+^ did not significantly affect the morphology of the coatings. Both the substituted and unsubstituted HA coatings were characterized by spherical particles, splats, and many irregular micro-pores, with some micro-cracks on the surface. The adhesive bond strength of the coatings was measured according to the ASTM C633 standard. All the samples showed cohesive failure, except the 5Sr,10Zn-HA, which exhibited a mixed mode of failure and adhesive behavior. Bioactivity was measured after the immersion of coatings in SBF for 1, 7, 14, and 21 days. The apatite layer formation was more significant on the pure HA and on the 10Sr,5Zn-HA coating, and less so on the 10Sr,10Zn-HA coating. The apatite layer, composed of agglomerated and spherical particles, completely covered the above-mentioned coatings. No apatite layer formation was detected on the 5Sr,10Zn-HA coating, even after 21 days of immersion. The solubility of pure HA and co-substituted HA coatings was investigated in an SBF solution. The co-substituted HA coatings showed a higher solubility compared to the pure HA, due to their low crystallinity and higher amount of amorphous phase. In particular, the 5Sr,10Zn-HA coating was found to be the most soluble among all the other coatings. The solubility profiles of Ca^2+^, Sr^2+^, and Zn^2+^ ions were characterized by an abrupt release during the early stage of immersion. The effect of Sr^2+^ and Zn^2+^ co-substituted HA coating on the MC3T3-E1 osteoblast cell proliferation was examined. The 10Sr,5Zn-HA and 10Sr,10Zn-HA coatings were characterized by a significantly higher cell proliferation than the pure HA, while the 5Sr,10Zn-HA coatings revealed cytotoxic effects with respect to the MC3T3-E1 cells. The morphology of the MC3T3-E1 cells adhered on the pure and co-substituted HA coating surfaces was investigated via SEM. All the surfaces were characterized by a flattened cell morphology with filopodia and pseudopodia extensions. The only exception was found for the 5Sr,10Zn-HA coating, which showed a poor cell attachment with round and stunt cells, evidencing a certain degree of cytotoxicity. Finally, the BSA absorption procedure was applied. The co-substituted HA coatings were characterized by a faster BSA adsorption (as a function of the degree of co-substitution) than the pure HA, due to a strong electrostatic force between Sr,Zn-HA and proteins. Among all the co-substituted HA coatings, the best results for BSA adsorption were obtained for the 10Sr,10Zn-HA coating.

Sr and cerium (Ce) were used to prepare a sulphonated poly(etheretherketone)/strontium, cerium co-substituted HA (S-PEEK/Sr,Ce-HA) composite coating on a 316L SS stainless steel substrate using a high-energy low-current DC (direct current) electron beam technique [91]. The presence of Sr and Ce in the S-PEEK/Sr,Ce-HA composite coating improved its corrosion resistance. The co-doping also enhanced bioactivity and biocompatibility of the coatings. The human osteosarcoma MG63 cell viability test was performed via an indirect method, in which the extracts collected from the immersion test were used for culturing the cells. The obtained results indicated that the co-substitution of Ca by Sr and Cu in HA not only efficiently offset the potential cytotoxicity of Cu, but also stimulated cell differentiation.

The electrodeposition technique was employed to prepare Zn,Cu co-substituted HA coatings on pure Ti [92]. The Zn,Cu-HA coating provided enhanced corrosion protection for Ti substrates, with excellent barrier properties. Co-substitution of HA with Zn^2+^ and Cu^2+^ led to a decrease in porosity, resulting in a denser coating. The ions were co-deposited homogeneously into HA films with no evident cracks or imperfections. Co-substitution of Zn^2+^ and Cu^2+^ ions had no significant effect on the adhesion strength of the Zn,Cu-HA coating to the Ti substrate, which was determined to be 9.4 MPa, slightly higher than that (8.1 MPa) for the pure HA coating. The smaller grain size of the Zn,Cu-HA coating boosted electron activity at the grain boundaries, which decreased I_corr_ and enhanced corrosion protection. The HA coating containing lower Cu^2+^ (1.22 wt%) and Zn^2+^ (1.02 wt%) contents showed good cytocompatibility and presented no cytotoxicity towards the osteoblast-like MC3T3-E1 cells. The release of Cu and Zn ions from the coatings was rapid within the first week, and osteoblast toxicity was not observed.

**Figure 2:**
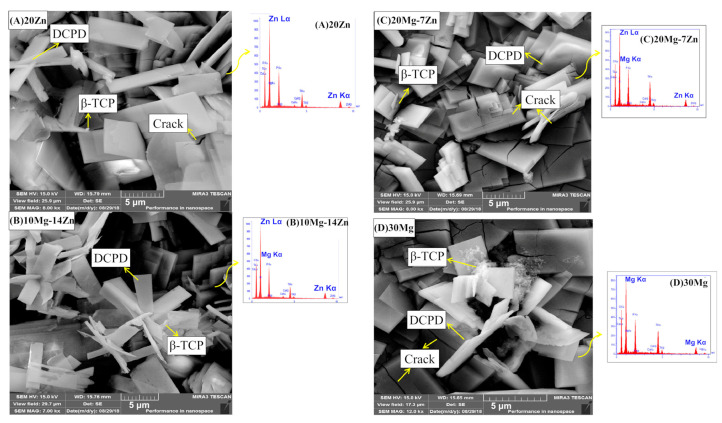
FE-SEM images with EDS pattern of the coatings: (**A**) 20Zn, (**B**) 10Mg-14Zn, (**C**) 20Mg-7Zn, and (**D**) 30Mg. Reprinted with modifications from “Surfaces and Interfaces, 22, Shahmohammadi, P.; Khazaei, B.A. Characterization of Zn/Mg-enriched calcium phosphate coating produced by the two-step pulsed electrodeposition method on titanium substrate, 2021” with permission from Elsevier.

Mg and Zn were included in CP coatings deposited using a two-step pulsed electrodeposition method on a Ti surface with a nanotube structure, and described in [93]. Samples with a different ratio of Mg and Zn, such as 20Zn-DCPD (dicalcium phosphate dehydrate) (20 mM of Zn), 10Mg,14Zn-DCPD (10 mM of Mg, 14 mM of Zn), 20Mg,7Zn-DCPD (20 mM of Mg, 7 mM of Zn), and 30Mg-DCPD (30 mM of Zn) were prepared and characterized. XRD analysis indicated that the addition of Mg and Zn led to a decrease in crystallinity and crystal size and to an increase in lattice parameters. SEM morphology investigation showed the presence of a re-precipitate phase, likely β-TCP, along with the predominant one—DCPD. The presence of Mg in the CP structure reduced the formation of the β-TCP phase and caused internal stresses, resulting in cracks on the coatings that acted as new nucleation sites. The increase in the Mg and Zn content in the coating led to a greater formations of cracks. Determination of protein uptake was carried out via FTIR analysis on the Mg,Zn-CP coatings immersed in 0.1 g/L of BSA for 24 h. The increase in the amount of Mg ions in the coating induced a higher protein adsorption intensity, with the 30Mg-DCPD sample having the highest uptake, as testified to by the intensities of the amide I bands. The authors concluded that this result can be attributed to the augmented surface area of the sample induced by the Mg addition. Field emission (FE)-SEM micrographs of 20Zn, 10Mg-14Zn, 20Mg-7Zn, and 30Mg coatings are reported in Figure 2.

A Mn and Sr co-substituted TCP coating was deposited via pulsed laser deposition on a zinc–lithium (Zn-Li) biodegradable alloy in [94]. The authors demonstrated via XPS analysis that both Mn and Sr were present in the coating in the form of ions, with no traces of metallic counterparts. XRD investigation confirmed the absence of signals attributable to metallic Mn and Sr. Structural XRD analysis revealed a modification of the β-TCP lattice parameters and lattice distortion upon the Mn and Sr substitution. This was attributed to the difference in ionic radii between Ca^2+^ (0.099 nm), and Mn^2+^ (0.046 nm) and Sr^2+^ (0.113 nm). FTIR patterns detected the presence of shifts of the PO_4_^3−^ bands due to the Mn and Sr substitutions. Moreover, when deposited on the Si substrate, the FTIR pattern of the Mn,Sr-TCP coating was characterized by convolution of the phosphate ν_4_ bending modes (549 cm^−1^ and 598 cm^−1^), suggesting a low degree of crystallinity of the coating. The film surface morphology, assessed via SEM, was characterized by a uniform nano-sized textured background with globular particles, with dimensions ranging from a tenth of a nm up to few microns, present on the surface, along with flattened splat aggregates.

Ag, Sr, Zn, and Mg were incorporated into a multi-doped bioactive calcium phosphate layer prepared via pulse current deposition onto surgical grade Ti alloy [95]. The metal ion-modified CP layer consisted of a mixture of needle-like agglomerates and rounded particles of some micrometers, whereas the pure CP coating, after surface treatment in 1M NaOH solution (performed to transform a mixture of CP phases into HA phase) consisted primarily of small needle-like particles of nanometric size. The changes in crystallite size were attributed to the substitution of Ca^2+^ ions by Mg^2+^, Sr^2+^, and Ag^+^ ions in the HA lattice. The passive current density of the multi-doped CP coating was higher than those of the uncoated substrate and the undoped CP coating, demonstrating that the ion-modified CP layers were less resistant to corrosion. The MG-63 human osteoblast-like cell studies revealed that the vitality percentage of cells seeded onto the pure CP coating was 74% with respect to the control, while the viability percentage of cells seeded onto the ion-modified coating was around 58%, after 3 days of culture. A possible explanation of the lower viability percentage given by the authors [95] is that an excessive amount of Ag can significantly impact the viability and metabolism of cells. The live/dead cell staining assessment revealed that the CP and ion-doped CP coatings were characterized by the presence of confluent and dense living cell layers with only a few dead cells, demonstrating their good biocompatibility.

A Si, Sr, and Ag co-doped HA/TN (Si,Sr,Ag-HA/TN) coating was prepared using the electrodeposition technique in [96]. Si, Sr, and Ag were incorporated into a HA matrix to form a Sr,Si,Ag-substituted HA/TN composite. The HA, Ag-HA, and Si,Sr,Ag-HA-coated samples displayed a strong HA(002)-preferred orientation and high crystallinity, with no lattice distortion or decrease in crystallinity due to the Si, Sr, and Ag dopants, as determined by the XRD. FE-SEM images of the HA and Ag-HA coatings revealed similar microstructural characteristics, such as a needle-like crystal morphology, although the Ag-HA crystals were finer than the HA crystals. The Si,Sr,Ag-HA/TN composite coating featured interconnected pores with an average diameter in the range of 10–20 µm. The Si,Sr,Ag-HA/TN coating had the roughest surface, with a roughness value of 1.23 μm, which promoted the MC3T3-E1 osteoblast cell adhesion and the expression of morphogenetic proteins. The HA and Ag-HA surfaces exhibited hydrophilic contact angles of 15.5° and 9.1°, respectively, while the Si,Sr,Ag-HA coating exhibited 100% hydrophilicity (0°). The release of Ag^+^ ions from the Si,Sr,Ag-HA coating was characterized by two-phase kinetics, with a quick release during the initial 5 days and a gradual release from the inner regions of the coating in the subsequent 6–14 days; whereas Sr^2+^ and Si^4+^ ions exhibited a slow and sustained release during the same time period. Si-Sr co-doping in the Si,Sr,Ag-HA coating boosted the growth of the MC3T3-E1 cells and mitigated the cytotoxicity of Ag ions.

## 3. Effects of Different Components in CP-Based Coatings

### 3.1. Polymeric Components

A sulphonated poly(etheretherketone)/Sr, Ce co-substituted HA (S-PEEK/Sr,Ce-HA) composite coating was prepared by electrodeposition on a 316L stainless steel substrate in [91]. FTIR and XRD studies demonstrated that the S-PEEK/Sr,Ce-HA composite was a mixture of S-PEEK and Sr,Ce-HA, without the formation of new phases due to the components’ interaction. The microstructure of the composite coating was leaf-like at 1 wt% of S-PEEK. At a concentration of 1.5 wt%, the S-PEEK/Sr,Ce-HA coating acquired an interconnected needle-like morphology. A further increase in the S-PEEK concentration up to 2 wt% led to a compact and uniform needle-like morphology with interconnected micropores. Studies of potentiodynamic polarization, based on electrochemical impedance spectroscopy, revealed that the S-PEEK/SrCe-HA (2 wt% S-PEEK) composite coating exhibited the highest corrosion resistance, compared to other investigated coatings and uncoated stainless steel specimens. Cell viability was examined using an indirect method, in which the extracts collected from the coatings immersion test were used for culturing of human osteosarcoma (HOS) MG63 cells. The cell viability findings demonstrated that the composite S-PEEK/Sr,Ce-HA (2 wt% S-PEEK) coating showed outstanding promotion of the cell viability (99.6%) by day 7, due to the presence of both Sr and Ce ions in the composite. The ability of the composite coating to generate apatite in SBF was evaluated on various days at 37 °C. After 21 days of immersion in SBF, the apatite deposition, with a morphology of a thick flower with pores between the petals, was observed by SEM. An accelerated growth of apatite on the S-PEEK/Sr,Ce-HA (2 wt% S-PEEK) composite coating deposited on the stainless steel substrate treated via high energy low current DC electron beam (HELCDEB) was attributed to the combined influence of Sr^2+^ and Ce^3+^ ions and S-PEEK present in the composite, as well as the enhanced anticorrosion performance of the HELCDEB-treated substrate. 

Minerals (M = Sr^2+^, Mg^2+^ and Ce^3+^)-substituted porous HA/Poly(3,4-ethylenedioxy pyrrole-co-3,4-ethylenedioxythiophene) (P(EDOP-co-EDOT)/M-HA) bilayer coatings were prepared on 316L stainless steel via electrochemical deposition and reported in [97]. XRD analysis of P(EDOP-co-EDOT)/M-HA coatings confirmed the bilayer nature of the P(EDOP-co-EDOT) coated with M-HA. The XRD patterns reveal that the M-HA coating had a substantial effect on the enhanced crystallinity of the P(EDOP-co-EDOT)/M-HA bilayer coating. SEM cross-section of the top M-HA coating revealed a porous network, while the inner P(EDOP-co-EDOT) layer and substrate were characterized by a continuous interface without pores or cracks along it. On the surface of the bilayer coatings, an increase in average roughness was found via AFM. The average roughness for the P(EDOP-co-EDOT)-3 compact layer was found to be approximately 350 nm, whereas that of the P(EDOP-co-EDOT)/M-HA bilayer coating was found to be 1.6 μm, suggesting a roughness increase attributable to the deposition of the HA layer. Investigation of mechanical properties revealed that the Vickers hardness value for the P(EDOP-co-EDOT)/M-HA bilayer was higher than that of the M-HA alone, demonstrating a good bonding strength between the M-HA and P(EDOP-co-EDOT) layers. Corrosion protection performance of the P(EDOP-co-EDOT)/M-HA bilayer-coated 316L SS specimen in Ringer solution was higher than that of the single component monolayers. ICP—atomic emission spectroscopy (AES) assay demonstrated that the investigated bilayer coating was characterized by a reduced metal ion release, resulting in a superior anticorrosion resistance. Thermogravimetric analysis (TGA) of the P(EDOP-co-EDOT)/M-HA registered a weight loss in the temperature range of approximately 100–120 °C, attributable to the hydrogen-bonding interaction between the trapped water and co-polymer chain, followed by a plateau, and a further, non-significant weight loss above 600 °C. 

A polyvinyl alcohol/cobalt(Co)-substituted HA (PVA/Co-HA) nanocomposite membrane was produced via electrochemical deposition on stainless steel and characterized in [98]. XRD study of the PVA/Co-HA nanocomposite membrane reported no peaks from Co-HA. Tensile testing revealed that the addition of Co-HA enhanced the tensile strength and ductility of the nanocomposite membrane. FTIR measurements revealed that the OH groups from Co-HA and PVA formed hydrogen bonds to restrict the mobility of molecular chains, related to the increased surface crystallinity. Using TGA and differential scanning calorimetry (DSC), the thermal properties of PVA and nanocomposites were evaluated. Co-HA was shown to slow the weight loss of the nanocomposite. SEM measurements were conducted after the TGA and DSC investigations. It was observed that, for temperatures higher than 380 °C, PVA uniformly coated the Co-HA particles, which resulted in a denser texture. Furthermore, XRD measurements carried out on heat-treated samples revealed that the addition of Co-HA significantly accelerated the PVA crystallization, as testified by the higher intensity of the PVA peak (101) in the PVA/-Co-HA composite. Results from a swelling behavior assay demonstrated that the PVA/Co-HA nanocomposite membranes showed good swelling properties: soaking in PBS for 1 min resulted in 150% membrane expansion. The capacity of samples to neutralize free radicals was tested by cyclic voltammetry. It was found that the PVA/Co-HA had a considerably better free radical capture capacity than the pure PVA or PVA/HA. Hydrophilicity testing reported the following contact angle results: PVA (52.0°), PVA-HA (48.5°), and PVA/Co-HA (41.5°), demonstrating that the addition of HA can effectively improve the hydrophilicity of the PVA surface, providing the best results for the PVA/Co-HA composite. After bioactivity testing of the PVA/Co-HA in SBF, SEM imaging revealed that the surface morphology of the investigated composite changed from a flat to a lamellar one. Finally, biocompatibility testing, using mouse-derived fibroblasts (L929) cells cultured in DMEM with the addition of the coatings’ extracts, demonstrated, after 72 h, that the viability of cells on the PVA/Co-HA was higher (approximately 112%) than on the control (100%) and on the pure PVA (approximately 97%), but did not differ significantly from the PVA/HA (approximately 105%) sample.

Polycaprolactone (PCL) electrospun fibers containing carbonated hydroxyapatite (CHA) partially substituted with selenite ions (SeO_3_^2−^) (Se-CHA/PCL) were prepared via electrospinning in [99]. Subsequently, Ag nanoparticles (AgNPs) were deposited on the fibers via pulsed laser deposition, resulting in (AgNPs@Se-CHA/PCL). Morphological and microstructural investigations carried out via FE-SEM revealed a homogeneous texture of fibers with diameters in the range of 0.38–0.6 μm and 0.85–1.12 μm, depending on the AgNP deposition time of 10 min and 20 min, respectively. Within the fiber network, it was possible to distinguish spherical shaped AgNPs with a wide range of diameters (from 80 nm to 140 nm). Furthermore, the increase in deposition time of AgNPs led to an increase in the surface roughness: 24.5 nm for 10 min of deposition and 35.0 nm for 20 min. Human osteoblastic HFB4 cells seeded on the micro-fibers coated with AgNPs@Se-CHA/PCL had a tendency to grow horizontally, following the fibers’ directions. The comparison between the samples characterized by different deposition time of AgNP allowed the authors of [99] to conclude that the AgNPs’ content and the roughness and hydrophilicity of the coated fibers’ surface contributed to the increase in the cell proliferation and affected the cells’ morphology.

Nanocomposite coatings made of polycaprolactone/fluoride-substituted HA (PCL/FHA) with various amount of FHA (10, 20 and 30 wt%) deposited on a Ti alloy substrate using an in situ sol–gel method were reported in [100]. Different morphologies and thicknesses of the coatings with various FHA contents were observed in SEM micrographs collected on pure PCL and PCL/FHA nanocomposite coatings. XRD confirmed an increase in the FHA crystallinity in the PCL/30 wt% FHA coating. FTIR superficial analysis showed a slight shift of the PCL characteristic peaks, particularly in the PCL/30 wt. FHA coating, likely caused by bonds between the FHA and PCL matrix. Using the water contact angle method, the hydrophilicity of pure PCL and PCL/FHA nanocomposite coatings was assessed. The hydrophilicity of the coatings increased with the increase in the FHA content due to the presence of hydroxyl groups. Indeed, the following contact angle results were obtained: PCL—88.3°, PCL/10wt%FHA—71.2°, PCL/20wt%FHA—65.5°, PCL/30 wt%FHA—51.2°. The adhesion test, performed according to the standard ISO 13779−4, showed that the coatings’ adhesive strengths increased with the increase in the FHA content. Pure PCL coatings had a smooth topography, while agglomeration of FHA was likely the cause of an increase in surface roughness of the composited coatings, followed by an increase in the FHA content: (PCL/20 wt%FHA—9.4 µm, PCL/30 wt%FHA—10.7 µm). The bioactive potential of samples coated with pure PCL and PCL/FHA nanocomposites was assessed using an SBF solution. All the PCL/FHA composite-coated samples showed apatite particle formation on their surfaces, which was attributed to the presence of FHA inducing the formation of apatite nucleation sites during the mineralization process with a typical “cauliflower” morphology. In contrast, there was no sign of apatite deposition on the surface of the pure PCL-coated sample during the first two weeks; after that, on day 21, a poor apatite deposition was registered. Therefore, composite-coated samples with higher FHA contents had higher reactivity in SBF solution, resulting in the dissolution of the coatings and the precipitation of apatite. Evaluation of electrochemical corrosion showed that the E_corr_ values of all the PCL/FHA-coated samples shifted to more positive values compared to the pure PCL coatings, and thus it was possible to conclude that the FHA particles dispersed throughout the PCL matrix allowed the slowing of the Ti alloy substrate corrosion. Human osteosarcoma MG63 cells viability assessment revealed that the hydrophobic properties of PCL reduced the cells’ viability, whereas the composite sample with 20 wt%FHA was characterized by an enhanced cell proliferation, up to 110% after 5 days of cell culture, as compared to approximately 90% for pure PCL and PCL/30 wt%FHA.

### 3.2. Biopolymeric Components

Chitosan Ag/HA composite coating (CSAg-HA) on TiO_2_ nanotube substrate was prepared via electrochemical deposition and investigated in [57]. FTIR study revealed a broadening of the band at 1035 cm^−1^, indicating the CS polymer presence and its interaction with the PO_4_^3−^ groups, whereas the carbonate ions in apatite were responsible for the bands at 1418–1468 cm^−1^ and 876 cm^−1^. The band at 3570 cm^−1^ corresponds to the hydroxyl group in HA. The interaction between the Ag ions and the NH_2_ groups of CS caused the characteristic peak of CO to shift to 1635 cm^−1^ upon the Ag ions inclusion. Surface morphology showed that, in both the HA and CSAg-HA coatings, needle-like HA crystals were formed; however, the CSAg-HA crystals were shorter and more densely packed than the HA crystals, suggesting that CS can promote the nucleation process with a consequent reduced crystal growth. Mouse calvarial MC3T3-E1 cell viability assay performed via the direct method indicated that more cells were present on the CSAg-HA sample than on the HA one: after 7 days of culture, cell viability on HA was 80%, while on CSAg-HA it was 90%. The results obtained by SEM demonstrated that the CS and Ag integrated with HA had no deleterious effect on the cell growth. Moreover, the integration of CS and Ag appeared appropriate for cell proliferation and differentiation. 

A composite coating of gentamicin antibiotic (Gent), natural polymer chitosan (CS), and HA (HA/CS/Gent) was successfully deposited on Ti via the electrophoretic deposition technique in [101]. FTIR investigation revealed that intermolecular hydrogen bonding between oxygen atoms from HA with hydroxyls or amino groups from chitosan was observed through a broad band at around 3281 cm^−1^. This band, registered in both the HA/CS and HA/CS/Gent coatings spectra, may account for the CS incorporation into the composites. Thermal analysis showed a thermal degradation process for both the coatings, starting from 220 °C and reaching its maximum at 293 °C, which consisted of the decomposition of chitosan chains into monomers followed by a series of processes, which led to the formation of cross-linked structure, more stable than linear chains. A weight loss, starting from 350 °C and reaching its maximum at 405 °C, occurred due to further CS or CS&Gent degradation, along with carbonate ion release from the HA structure as CO_2_ molecules. SEM investigation revealed that CS affected the coatings’ morphology, since it helped to create a homogeneous, porous, fracture-free surface on Ti plates due to a strong interfacial interaction between the HA particles and the CS matrix. Adhesion strength results, obtained according to the ISO 2409 standard test, evidenced the HA/CS/Gent coating’s good adhesion, without any issues related to detachment or flaking-off, thanks to the formation of intermolecular hydrogen bonds between the HA and CS components, evidencing that CS acted as an adhesive between the HA and Gent.

A halloysite nanotube-reinforced metal ion-incorporated (M = Zn and Mg) HA- chitosan composite coating on Ti-6Al-4 alloy (HNT-CS-MHA-Ti) was prepared via electrodeposition in [102]. SEM morphology investigation and cross-section analysis were carried out, and the obtained images revealed that the substrate surface was entirely and homogeneously covered by a developed composite coating thicker than 50 μm, with no fractures or cavities. FTIR analysis detected the presence of the characteristic bands of amide II and amine group from CS, while another characteristic peak of CS at 1643 cm^−1^ of amide I disappeared, leading to a hypothesis that electrostatic interaction and hydrogen bonding between NH^3+^ from CS and PO_4_^3−^ from HA may have occurred, while the vibration peak of N-H shifted to the 1578 cm^−1^ band with a decreased intensity, indicating a possible interaction between the CS and HNT-MHA composites. XRD analysis revealed a shift in HA diffraction peak positions towards lower angles, compared to conventional XRD patterns, according to authors [102] This shift is a result of HA compression through the interfacial interaction with the contracting polymeric matrix. Electrochemical analysis demonstrated that the use of CS in the composite coatings improved their corrosion resistance, providing an excellent protection against corrosive environments, as indicated by the higher E_corr_ value. Finally, the adhesive strength of the HNT-CS-MHA composite coating on Ti alloy (22.5 MPa) was found to be higher than that of the HNT-MHA (20.1 MPa), CS-MHA (16.2 MPa), and MHA (16.9 MPa) coatings.

Ag,Sr-substituted HA/chitosan (CS/Ag,Sr-HA) coatings on 316L stainless steel were prepared via electrophoretic deposition in [103]. FTIR analysis revealed a minor shift in the NH peak of CS as a result of the hydrogen bonds established between the HA and chitosan. SEM surface studies revealed the homogenous nature of the composite coatings with uniformly distributed Ag,Sr-HA particles, thanks to the presence of CS in the electrolyte suspension. Wettability tests revealed a good hydrophilic behavior of the coatings. Indeed, the obtained contact angle values were the following: stainless steel substrate—86°, chitosan on substrate—78°, composite coating—50°. The adhesion strength was determined by the tape test, according to the ASTM D3359-97 standard. As determined by the test, 5% of the coating was delaminated from the substrate. The adhesive strength results indicated that chitosan acted as a cohesive phase, able to bind and embed HA particles into the composite matrix. The bioactivity of the CS/Ag,Sr-HA composite coatings was evaluated via immersion in SBF for 3 days. SEM images collected after the immersion period showed that a “cauliflower-like” apatite structure with nano-porous and highly dense apatite crystals was formed on the composite coating surface, attesting its good bioactivity.

Ce-doped HA/collagen coatings deposited via the biomimetic method using a modified supersaturated calcification solution, additionally containing a Ce source and collagen, were reported in [104]. The presence of collagen fibrils and HA was confirmed by FTIR analysis. The peak at 1332 cm^−1^ was a result of the electrostatic interaction between Ca^2+^ ions from HA and the COO groups from collagen. SEM analysis of the surface morphology revealed the presence of the HA crystals (with sizes less than 100 nm) attached to the collagen fibrils. The SEM-EDS investigations confirmed the presence of Ce in the coating and determined its Ca/P ratio = 1.67, characteristic for HA. 

Lanthanum (La), terbium (Tb)-substituted HA/biopolymer duplex (chitosan (CS) and casein (CA)) multi-layer coatings on anodized Ti were developed via electrodeposition in [105]. The anodization of the Ti substrate led to the formation of TiO_2_ nanotubes (TN). The La,Tb-HA layers (with constant content of La and different content of Tb) were deposited on the CS-CA/TN composite coatings. The latter exhibited sharp diffraction peaks corresponding to the CS and CA polymers. The detected sharp diffraction signals of CS and CA indicated their high crystallinity, as a result of strong hydrogen bonding between hydroxyl groups of CA and amine groups of CS. SEM study of surface morphology evidenced the presence of a multilayer structure: uniform, interconnected, granular La,Tb-HA layer on porous CS-CA layer on TN. The multi-layer coating structure with 16–20 µm of thickness enhanced the hardness of the final coating, 8 GPa being the highest value. The results of potentiodynamic polarization experiments indicated that the multi-layer coatings were characterized by a higher corrosion resistance with respect to CS-Ca/TN and TN alone. The best results for the human osteoblast MG 63 cell viability and live/dead staining assay were achieved for the multi-layer composite coatings with (0.05 mol of La and 0.075 mol of Tb). 

A Zn-HA coating impregnated with phosphatidylethanolamine (PE)—PE/Zn-HA—was deposited via cathodic deposition on a Ti surface and reported in [72]. It was found that PE influenced the wettability of the surface, the PE/Zn-HA surface contact angle (56°) being more hydrophilic than the control Zn-HA surface (63°). The adhesion strength test revealed a better adhesion strength of the PE/Zn-HA to the Ti substrate (9.5 MPa) compared to that of the control Zn-HA surface (8.2 MPa). The incorporation of the PE component into the Zn-HA coating resulted in an enhanced osteoblast survival: PE/Zn-HA—120%, Zn-HA—100% (used as control).

Sr, Mg, and Zn-substituted HA/gelatin (M-HA/Gel) nanocomposite coating prepared by pulsed electrodeposition on electron beam-treated Ti was reported in [106]. The obtained results accounted for a complexation of M^2+^ ions (M^2+^ = Ca^2+^, Sr^2+^, Mg^2+^, and Zn^2+^) with HA and gelatin, resulting in the development of partial bonds between the M^2+^ cations and gelatin. The M^2+^ and gelatin complex molecules combined with PO_4_^3−^ ions to generate the M-HA/Gel composite. FTIR measurements of the surface proved that carboxylate (COO) and amino (–NH–) groups in gelatin can establish bonds with the P–O and OH^−^ groups of M-HA. The presence of the C–H bond of the gelatin in the composite was responsible for the peaks at 2923 and 2852 cm^−1^, indicating that gelatin interacted with M-HA. Two bands at 985 and 937 cm^−1^, attributable to P–O–C aliphatic stretching and P–N–C stretching, provided the evidence for binding of phosphate ions to gelatin. The XRD peak at 20° supplied a further confirmation of the binding of gelatin with HA. The coating material was crystalline, and the gelatin concentration did not influence the HA crystallinity. The morphological characteristics obtained by SEM were dependent on the gelatin concentration in the composite, varying from a dumbbell-shaped structure with some incomplete surface coverage for (1 wt% gelatin) M-HA/Gel coating, to a dense flower petal-like morphology for (4 wt% gelatin) M-HA/Gel coating. The adhesion strength of the M-HA/Gel composite coatings was approximately 23 MPa. Potentiodynamic polarization measurements conducted in SBF solution demonstrated that the M-HA/Gel nanocomposite coating with 3 wt% of gelatin exhibited superior anticorrosion performance. Live/dead MC3T3-E1 mouse osteoblast cells evaluation assay established that cells preferred expanding on the developed nanocomposite surface. After 7 days of cell culture, their proliferation on the nanocomposite was significantly higher than on the control, mainly due to the presence of mineral ions and gelatin.

Ag-doped HA (Ca_9_._95_Ag_0_._05_(PO_4_)_6_(OH)_2_) with natural polymer lignin (Ag-HA/Lig) composite coatings were deposited via electrophoretic deposition on Ti in [53]. Various concentrations of Lig (0.5, 1 and 10 wt%) were used. The investigation of the coatings after their immersion in SBF for 7 days was performed, providing evidence on apatite deposition occurred upon the immersion. FTIR analysis detected a peak at 1101 cm^−1^ due to P-O stretching of phosphate group and a shift of the peak at 635 cm^−1^, attributable to the OH group of HA, accounting for the formation of intermolecular hydrogen bonds between HA and Lig. Moreover, the peaks at 1642, 1460 and 1424 cm^−1^ were attributed to the CO_3_^2−^ groups, indicatively arising from carbonated apatite layer formed upon the immersion in SBF. The XRD diffractograms of Ag-HA/Lig coatings revealed that after the immersion period, the (002), (211) and (300) HA reflections shifted towards higher angles. SEM micrographs revealed that the coating with 1 wt% of Lig was characterized by a homogeneous, fracture-free surface. On this smooth surface, apatite crystals with a plate-shape morphology were observed. The results obtained by FTIR, XRD, and SEM investigations allowed authors to conclude that the Lig component provided nucleation sites for apatite growth and, therefore, enhanced the bioactive potential of the composite coating. Cytotoxicity evaluation of the composite coating was conducted via MTT test on human peripheral blood mononuclear cells. Cell survival outcomes indicated that the coating with 1 wt% Lig was considered non cytotoxic (89% of viable cells), whereas the coating with 10 wt% Lig was characterized by a slightly cytotoxic behavior (76% of viable cells).

### 3.3. Inorganic-Based Materials Components

Ag-substituted HA/TiO_2_ composite coating deposition via plasma electrolytic processing on a Ti surface, pretreated by plasma electrolytic oxidation to form a TiO_2_ layer, was outlined in [52]. The XRD pattern revealed that the coating contained both TiO_2_ and Ag-HA phases, and a new phase of titanium oxide phosphate (Ti_5_O_4_(PO_4_)_4_) with a characteristic diffraction peak at 2θ = 28.8°. SEM micrographs revealed that the TiO_2_ surface was characterized by a homogeneous porous morphology with a pore size of 2–4 μm. SEM images collected from the Ag-HA/TiO_2_ composite coating revealed that Ag-HA was present in the form of particles evenly dispersed and adherent to the TiO_2_ surface layer, with some particle agglomerates trapped within the pores. Electrochemical corrosion testing conducted in SBF solution showed that TiO_2_ helped to improve the corrosion resistance of the Ag-HA/TiO_2_ composite coating; in particular, the polarization resistance of uncoated Ti was 9.8 × 10^4^ Ωcm^2^, while that of the composite Ag-HA/TiO_2_-coated Ti was significantly higher—1.2 × 10^6^ Ωcm^2^. This finding proved the semi-insulating behavior of TiO_2_.

A graphene oxide(GO)/oxidized carbon nanofiber (O-CNF)/mineralized HA hybrid composite material (Mg,Zn-HA/O-CNF/GO) was prepared via chemical synthesis in solution [107]. FTIR analysis reported the presence of all the characteristic bands for the Mg,Zn-HA/O-CNF/GO and confirmed the successful synthesis of the composite. The XRD pattern of the Mg,Zn-HA/O-CNF/GO composite was characterized by broad peaks, indicating the formation of a low crystalline composite. Moreover, the GO peak shifted towards lower angles, indicating the interaction between the two components. FE-SEM studies revealed the presence of a homogeneous surface with a hexagonal structural arrangement of the Mg,Zn-HA/O-CNF/GO material. Vickers microhardness values of 330 HV for Mg,Zn-HA alone, 425 HV for Mg,Zn-HA/GO composite, and 468 HV for Mg,Zn-HA/O-CNF/GO composite were obtained. The strong electrostatic interactions between Ca^2+^ ions present in Mg,Zn-HA and the carboxyl and carboxylate groups of the O-CNF/GO hybrid material boosted the interfacial bonding strength, hence enhancing the hardness characteristics. An in vitro biocompatibility MTT assay revealed a 99% viability for human osteoblast MG63 cells exposed to the M-HAP/O-CNF/GO composite material, after 5 days of incubation, evidencing that the inclusion of both the components (GO and O-CNF) showed no negative effect on the cells.

Single-layer Si and double-layer MgO/Si coatings were fabricated on Mg/27.5 wt% HA nanocomposite sintered pellets via the milling-multi steps pressing-sintering powder metallurgy method and reported in [108]. The FE-SEM morphology investigation revealed that the outer layers of the Si- and MgO/Si-coated samples were rough, with micropores, and mainly composed of microparticles with irregular morphologies. There was no discernible discontinuity between the coatings and nanocomposite substrate, indicating that the coating and substrate were strongly bonded, which was also confirmed by SEM cross-sections measurements. Adhesion strength experiments reported a value of 26 MPa for the Si monolayer coating and 32 MPa for the MgO/Si double layer coating, indicating the importance of MgO as an interlayer in enhancing the coating’s adhesion to the substrate. Compressive strength experiments were conducted according to the ASTM E9-09 (2009) standard on the complete set of samples (uncoated Mg/27.5HA substrate, Si-coated and MgO/Si-coated substrate) before and after immersion in SBF for 28 days at 37 °C. The obtained results indicated a higher compressive strength performance before immersion for the Si-coated and MgO/Si-coated samples, which were characterized by 236 and 228 MPa, respectively; whereas, for the uncoated Mg/27.5HA, a compressive strength value of 228 MPa was obtained. The lower performance of the MgO/Si coating, with respect to the Si one, was attributed to the formation of microcracks on the surface of the former. The same test was conducted after 28 days of immersion in SBF and, for all the samples, the deterioration of performance was reported: 186, 174, and 161 MPa for MgO/Si-coated, Si-coated, and uncoated Mg/27.5HA, respectively. The lower performance was attributed to the degradation of Mg in the SBF solution environment. The corrosion rate resistance of the MgO/Si-coated composite samples was higher (0.18 mm/year) than that of the Si-coated samples (0.65 mm/year), even though both were more resistant compared to bare Mg/27.5HA (4.28 mm/year). Due to the dissolution of Mg, the uncoated Mg/27.5HA nanocomposite matrix was severely attacked, whereas the developed coatings protected the substrate against harsh solutions. FE-SEM micrographs showed that 14 days of immersion in SBF were sufficient to reduce the number of pits and deep cracks on the surface of the double layer MgO/Si nanocomposite. In vitro biocompatibility evaluations were assessed via the MTT test on murine osteoblastic MC3T3-E1 cells. The cell viability was evaluated after 9 days of culture with the extract of each sample. It was observed that the MgO/Si-coated sample showed the highest biocompatibility (95% of viable cells), followed by the Si-coated sample (78% of viable cells) and Mg/27.5HA (75% of viable cells) substrate. Moreover, the cell morphology investigation carried out via SEM revealed that the cells seeded on the Si coating surface presented an abnormal spherical shape, while on the MgO/Si nanocomposite surface, the cell morphology was well preserved.

HA/GO composite coatings on 316 L SS substrates, prepared using the dip and spin coating deposition technique, were described in [109]. Morphology investigations demonstrated the bonding capability of rod-shaped HA particles to the surface of graphene oxide nanosheets, leading to the reinforcement of the HA/GO matrix. In addition, the incorporation of GO resulted in a size reduction in the HA particles from 41 to 26 nm, these values being in good agreement with the sizes determined from XRD data using the Scherrer equation. GO sheets contain negatively charged functional groups on their surface, such as carboxylic (-COOH), carboxyl (-C=O), epoxy (-C-O), and hydroxyl groups (-OH), which likely acted as nucleation sites, attracting positively charged Ca^2+^ in the HA structure. The integration of GO promoted the biomineralization process in SBF, and higher mineral precipitation density was registered concomitantly to the higher GO content in the composite coating. SEM images collected from the HA/GO(3 wt%) sample revealed a dense layer of precipitate on the surface with fewer cracks and gaps, compared to the HA/GO (1 wt%) and the HA/GO (2 wt%) coatings. Measurements of Vickers hardness revealed that the addition of GO to the HA matrix at concentrations ranging from 0% to 3% enhanced its hardness from 2.6 GPa to 4.2 GPa, respectively. Potentiodynamic polarization and electrochemical impedance spectroscopy were used to investigate the corrosion resistance of the prepared coatings. The Tafel polarization plots showed that the increase in the GO concentration led to an enhanced corrosion resistance efficiency. The highest corrosion resistance was achieved for the HA/GO(3 wt%) coatings. The charge transfer resistance for all the coated substrates was found to be increased thanks to their barrier effect, compared to the non-coated substrates, and the charge transfer resistance values showed an increasing trend with the increase in the GO content. Biocompatibility was evaluated via the MTT assay using osteoblast MG-63 cells. The cell viability was measured at different concentrations of the extracts from pure HA and HA/GO@3%. The obtained results showed that the addition of GO to HA showed good biocompatibility with minimal cell death, similar to the pure HA, except in the highest concentration of the extract (500 μg/mL), where up to 22% of dead cells was registered.

A MgO/Tb,Eu (europium)-substituted HA double layer coating on borate passivated 316L SS prepared via electrodeposition was described in [110]. FTIR analysis verified that MgO was successfully applied as a coating on a Tb,Eu-HA layer. Several bands, attributable to OH- and phosphate groups, were indicative for the HA layer, while the peak at 667 cm^−1^ was attributed to the MgO stretching. XRD investigation confirmed the presence of HA and MgO phases, both characterized by high crystallinity. Corrosion resistance analysis revealed that the MgO/Tb,Eu-HA double layer coating had the best corrosion resistance performance, and also higher impedance values than the uncoated and single layer-coated samples. This demonstrated that the addition of MgO to Tb,Eu-HA coatings on a passivated SS substrate improved both the corrosion behavior and resistance. The adhesion strength of MgO/Tb,Eu-HA coatings (14.2 MPa) was higher than that of both the single layer samples (10.5 MPa and 12.4 MPa for HA coating and MgO coating, respectively). The Vickers micro-hardness test confirmed that the synergistic effect between the two layers induced an increase in the coating’s hardness (HV): 362.5 for MgO/Tb,Eu-HA, 316.6 for HA and 284.5 for MgO. Cytocompatibility was assessed via the MTT test on human pre-osteoblastic MC3T3-E1 cells. Cells cultured for 7 days with the extract of MgO/Tb,Eu-HA at concentration of 100 μg/mL exhibited approximately 95% viability. The live/dead staining assay was used to investigate the cells on the coatings at 1, 3, 5, and 7 days of incubation. The MgO/Tb,Eu-HA coating showed a good attachment and spreading morphology of live cells after 7 days of culture, with respect to the HA and MgO monolayers alone. 

### 3.4. Nanotube-Shaped Materials Components

**Figure 3:**
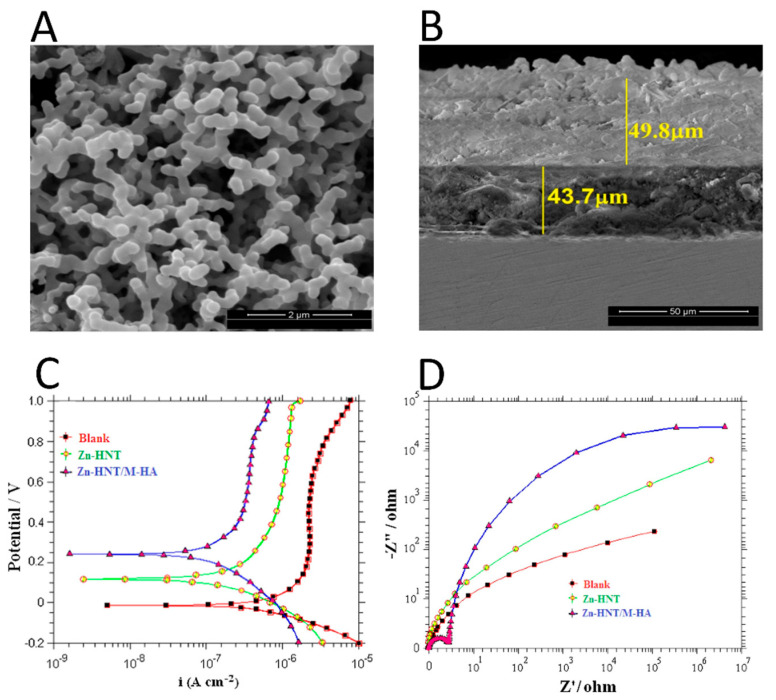
(**A**) SEM micrograph of *ZnHNT/Sr,Sm-HA* bilayer-coated Ti6Al4V (**B**) Cross-section morphology of ZnHNT/Sr,Sm-HA bilayer coating on Ti6Al4V. (**C**) Potentiodynamic polarization curves of blank (Ti6Al4V) (black squares), Zn-HNT-coated Ti6Al4V (orange circles), and Zn-HNT/Sr,Sm-HA bilayer-coated Ti6Al4V (blue triangles) in SBF solution. (**D**) Nyquist plots of blank (Ti6Al4V) (black squares), Zn-HNT-coated Ti6Al4V (orange circles), ZnHNT/Sr,Sm-HA bilayer-coated Ti6Al4V (blue triangles) in SBF solution. Reprinted with modifications from “Colloids and Surfaces A: Physicochemical and Engineering Aspects, 511, Chozhanathmisra, M. et al., Development of zinc-halloysite nanotube/minerals substituted hydroxyapatite bilayer coatings on titanium alloy for orthopedic applications, 357–365, 2016” with permission from Elsevier.

A Zn-halloysite nanotube(Zn-HNT)/Sr,Sm (Samarium)-substituted HA bilayer coating was deposited via electrodeposition on a Ti6Al4V substrate and characterized in [111]. By means of FTIR and XRD analysis, peaks of both Sr,Sm-HA and Zn-HNT phases were distinguished. SEM micrographs demonstrated that the Zn-HNT/Sr,Sm-HA bilayer coating and the Sr,Sm-HA monolayer coating were both characterized by a porous rod-like structure, but in the bilayer coating it was more uniform and compact. Cross-section analysis of nanotubes with the ZnHNT/Sr,Sm-HA bilayer coating allowed for quantification of the thickness of the Sr,Sm-HA top layer (50 μm) and the Zn-HNT bottom layer (44 μm). Furthermore, the bilayer structure was homogeneous and characterized by the absence of cracks or discontinuities. Corrosion resistance testing was assessed via potentiodynamic polarization. The obtained results revealed a superior anticorrosion performance for the Zn-HNT/Sr,Sm-HA bilayer coating, as testified by the E_corr_ characteristic values. Moreover, study via electrochemical impedance spectroscopy confirmed that the corrosion resistance of the Zn-HNT/Sr,Sm-HA bilayer coating (65.3 × 10^5^ Ωcm^2^) was significantly improved, compared to the Sr,Sm-HA coating (5.0 × 10^5^ Ωcm^2^). Cell viability testing using HOS MG63 cells was assessed for the Zn-HNT/Sr,Sm-HA bilayer coating. The cells were cultured in the extract at a concentration of 100 μg/mL, and the Sr,Sm-HA was used as the control. The obtained results showed a cell viability of 99.7%, which suggested that the addition of the Zn-HNT component did not significantly affect the biocompatibility. In addition, optical microscopy revealed the presence of live cells after 4 days of culture, characterized by the same morphology as the control group, confirming the non-toxic nature of the bilayer coating. Results from SEM investigation and electrochemical study of the ZnHNT/Sr,Sm-HA bilayer coating are presented in Figure 3.

Calcium silicate(CS)/Ag-doped HA coatings (Ag-HA/CS) deposited on TN and Ti substrates via electrodeposition were reported in [83]. The adhesion strength test was performed in accordance with the international standard (ASTM F1044-05) on the AgHA/CS deposited on Ti and Ag-HA/CS/TN deposited on Ti. Comparison of adhesive strength values of Ag-HA/CS/TN/Ti (15.2 MPa) with AgHA/CS/Ti (7.2 MPa) proved a significant increase in the adhesive performance due to the presence of TN, which enhanced the cohesive force and bonding between the Ag-HA/CS top layer and the Ti substrate. Electrochemical studies were conducted on Ag-HA/CS/TN/Ti, Ag-HA/Ti, and the bare Ti substrate. The values of E_corr_ from Tafel curves and of corrosion resistance from electrochemical impedance spectroscopy study revealed a higher corrosion resistance of the Ag-HA/CS/TN/Ti composite. This result allowed the authors to deduce that the presence of TN in conjunction with CS enhanced the anticorrosion performance. This experimental fact was explained by the “open-defects mechanism”, where the deposition of uncompressed HA on a substrate allowed free passage of species through open-defects, resulting in easy oxygen access to the substrate surface and self-regeneration of a protective oxide layer. Wettability studies were performed on the Ag-HA/CS/TN/Ti and Ag-HA/CS/Ti coatings and on TN/Ti. The TN/Ti was shown to be extremely hydrophobic (141°), while the Ag-HA/CS/TN/Ti and Ag-HA/CS/Ti shared the same hydrophilic behavior (0°). This significant change in the wettability performance was attributed to the Ag-HA top layer. The bioactivity assay attested that 5 days of soaking in SBF resulted in appearance of a well-formed apatite layer with typical spherical shape particles. Its formation was more significant for the Ag-HA/CS/TN/Ti than for the HA/Ti, indicating a higher bioactivity of the former due to the negatively-charged Si-OH and Ti-OH functional groups present in the CS and TN components, respectively, interacting with the positively-charged Ca ions in the SBF and eliciting the deposition of Ca compounds. 

A Cu-HA coating deposited via the electrophoretic method on a nanotubular TiO_2_ layer (obtained by anodization process) on a Ti13Zr13Nb alloy was described in [75]. A comparative XRD investigation was carried out for the Cu-HA/TN/Ti and Cu-HA/Ti coatings. The peak positions of crystalline HA were influenced by the presence of a nanotubular surface, as testified by a peak shift towards higher angles, attributed to the Cu-HA/TN/Ti sample. SEM morphology investigation was conducted on coatings characterized by different deposition times of the Cu-HA component. The obtained images revealed that, after 1 min of deposition, the sample surface was characterized by islets of non-homogeneously distributed HA agglomerates of spheroidal-shaped particles. The TN layer was visible in uncovered or less covered zones. The images collected after 2 min of deposition showed a more homogeneous layer of HA, though agglomerates were still present. The average coating thickness increase from 1.3 μm to 2.7 μm, for 1 min and 2 min deposition time, respectively, was registered. AFM topography was evaluated for the Cu-HA/TN/Ti and Cu-HA/Ti samples, showing that the average surface roughness of the Cu-HA/TN/Ti (0.7 μm) was lower than that of the Cu-HA/Ti (0.9 μm). Mechanical tests on the investigated coatings indicated that the presence of nanotubular oxide layer had no significant effect on the nanoindentation results, but did have an effect on the adhesion strength. The adhesion strength test values of 70.0 mN for Cu-HA/Ti and 80.6 mN for Cu-HA/TN/Ti were obtained, suggesting that the TiO_2_ nanotube layer had an anchoring effect. A wettability test was performed on the Cu-HA/TN/Ti, Cu-HA/Ti and TN/Ti samples. The TN surface was characterized by a contact angle of 67°, confirming the hydrophobic nature of the nanotube layer. The Cu-HA/Ti was characterized by a contact angle of 31°, while the Cu-HA/TN/Ti showed a value of 15.2°, being the most hydrophilic, and revealed a synergistic effect between the top Cu-HA and the intermediate TN layers. 

A Si, Sr, Ag multi-doped HA/TiO_2_ coating (Si,Sr,Ag-HA/TN) was deposited via electrochemical deposition on Ti in [96]. FE-SEM analysis of the top HA and Ag-HA surfaces revealed that both possessed similar dense microstructural characteristics, being composed of needle-like crystals. SEM cross-section images of the Si,Sr,Ag-HA/TN composite coating revealed the existence of 3D linked pores with diameters in the range of 10–20 μm. No visible discontinuity was noticed between the outer and inner layers of the composite coating. Electrochemical corrosion resistance analysis was carried out via impedance and polarization studies for all the coated samples (HA/TN, Ag-HA/TN and Si,Sr,Ag-HA/TN) and the uncoated Ti. All the coated samples were characterized by an improved corrosion resistance with small differences in E_corr_ values among them. These results were interpreted as a consequence of strong bonds between the Ti substrate and the composite coating thanks to the TN layer, enhancing the corrosion resistance and durability of Ti in a physiological environment. According to the adhesion strength evaluation test, the bonding strength measured for the Si,Sr,Ag-HA/TN bilayer (16.9 MPa) was more than doubled with respect to that of the HA/Ti monolayer (7.9 MPa). This result was interpreted as an interlocking process between the Si,Sr,Ag-HA and TN layers. 

Cu-substituted HA/functionalized multi-wall carbon nanotube (Cu-HA/MWCNT) composite coatings were deposited on 316L SS via spray pyrolysis in [77]. FTIR and XRD phase analyses confirmed the presence of the HA and MWCNT phases with no formation of additional compounds. FE-SEM and TEM morphology investigations revealed that nanosized spherical-shaped HA particles adhered to the MWCNT walls. Analysis of potentiodynamic polarization performed for the Cu-HA/MWCNT and HA/MWCNT revealed an improved corrosion resistance of both the composite coatings compared to the 316L SS substrate, these results being interpreted as a consequence of the adequately thick (70 µm), dense, and homogeneous Cu-HA/MWCNT composite coating with a smooth and compact surface.

## 4. Effect of Ion-Doped Coatings on Specific Bacteria Strains

### 4.1. Pseudomonas Aeruginosa 

The antibacterial activity of Ag-containing HA coatings on Ti against Gram-negative *Pseudomonas aeruginosa* (*P. aeruginosa*) and other bacteria strains was investigated in [112]. The evaluation of antibacterial activity was performed by counting the colony-forming units (CFU) based on SEM images. For the Ag-containing HA coatings with 20 ppm of Ag ions, the colony number decreased from 1730 to 0. It was shown that Ag ions had a significant effect on the morphology of the bacteria, leading to the cytoplasm membrane detaching from the bacteria wall, the appearance of a remarkable electron-light region in the bacteria cells, and the occurrence of condensed DNA molecules in the center of the electron-light region (SEM images). EDS microanalysis detected the presence of Ag in dense granules on the coating’s surface, and it was hypothesized that the bacteria inhibition or death was caused by Ag interacting with sulfhydryl groups present in enzyme proteins, drastically reducing the number of bacteria colonies.

Ag-containing HA coatings deposited on Ti at various concentrations of Ag and their antibacterial activity were reported in [47]. The antibacterial effect was assessed after 48 h of incubation by counting the CFU. The coatings exhibited good antibacterial ability against *P. aeruginosa*. In particular, 2.4% bacteria viability was registered for the lowest Ag content (0.7 wt%), and 0% for the highest Ag content (4 wt%). The Ag ion release in culture solutions was investigated, and its concentration was found to be 2.27 ppm for the sample with the highest Ag content (4 wt%), after immersion for 49 days in SBF. The mechanism of inhibitory action of Ag ions on bacteria was believed to consist of the interaction of Ag with thiol groups in proteins, or lie in affecting DNA and destroying its ability to replicate.

The antibacterial properties of Ag-doped HA films with Ag concentration of 0.5 and 1.5 wt% deposited on Ti were described in [60]. The bacteria’s adhesion and response to coating release were investigated. The number of dead *P. aeruginosa* bacteria on 0.5 wt% Ag-containing coatings increased modestly (16%) after 8 h and significantly (95%) after 24 h. In the samples containing 1.5 wt% of Ag, a larger proportion of dead *P. aeruginosa* cells (18 and 97%) was detected after 8 and 24 h of culture, respectively. Minimal changes for both the coatings occurred after 48 h of culture. An adhesion experiment carried out on bare Ti revealed that *P. aeruginosa* bacteria entirely colonized the Ti surface after 32 h, as evidenced by SEM. Gram-negative *P. aeruginosa* did not form massive three-dimensional colonies, as with Gram-positive *S. epidermidis*, but released extracellular polymers for protection against environmental stresses and antibiotics, allowing biofilms to grow on the Ti substrate surface. In the film delamination release experiment, in a media suspension of Ag-containing delaminated samples, *P. aeruginosa* bacteria exhibited a minimal growth after 7 h, in the case of the 0.5 wt% Ag samples; and no growth, in the case of the 1.5 wt% Ag samples. Ag appeared to have a greater effect on Gram-negative bacteria (*P. aeruginosa*) than on Gram-positive bacteria (*S. Epidermidis*).

A Ti-doped HA nanoparticle material containing 7.3 wt% of Ti deposited on a polyethylene (PE) sheet and irradiated by UV for 30 s was reported in [113]. The antibacterial efficacy was investigated through a viability assay, by comparing the results obtained from Ti-HA/PE samples with and without the UV irradiation. Without the UV treatment, a 50% decrease in the number of *P. aeruginosa* was registered compared to the control sample; whereas, for the UV irradiated Ti-HA-coated sheets, up to 75% dead bacteria was detected. It was suggested that the results obtained for the UV-irradiated samples might be caused by the photocatalytic activity of Ti contained in the HA nanoparticles.

The antibacterial activity of a Mn-containing tricalcium phosphate (Mn-TCP) coating with 2.3 wt% of Mn on Ti against various bacteria strains, including *Escherichia coli* (*E. coli*), *Enterococcus faecalis* (*E. faecalis*), and *P. aeruginosa*, was investigated in [114]. The antibacterial activity was assessed via the evaluation of the width of the bacterial culture growth suppression zone. The results showed that the Mn-TCP powder had an inhibition rate of 7% against *E. coli*, 20% against *E. faecalis*, and 15% against *P. aeruginosa*. In comparison, the unsubstituted TCP powder had an inhibition rate of only a few percent against the same bacteria strains. It was explained that the relatively low degree of antibacterial activity was likely due to the Mn state in the coatings. The EPR analysis showed that Mn was present in the form of metallic clusters instead of free ions, which were probably less soluble and, therefore, less effective against bacteria.

The antimicrobial properties of Ag-substituted TCP nanocoatings on a Ti alloy were reported in [55]. After 24 h of incubation, the effect of TCP and Ag-TCP coatings on planktonic growth and bacterial adhesion was determined by measuring the optical density of a bacterial suspension and of bacteria adhered on the coatings’ surface. The presence of Ag in the coating decreased the optical density of the *P. aeruginosa* suspension by 18.5%, compared to the pure TCP. The antibacterial effectiveness of the pure TCP coating was ascribed to its morphological features, while the Ag incorporation induced a more significant antimicrobial effect. Moreover, the bacteria adhesion activity was tested, and it was found to be approximately 30% for the Gram-negative *E. coli* and *P. aeruginosa*, and approximately 40% for the Gram-positive *S. aureus* and *E. faecalis*. It was concluded that the Gram-negative *E. coli* and *P. aeruginosa* were more sensitive to Ag than the Gram-positive *S. aureus* and *E. faecalis*. 

The antibacterial properties of single-doped Ce-HA (1.25 mol% Ce) and co-doped Ce/Si-HA coatings (1.25 mol% Ce and 5 mol% Si) on Ti were described in [115]. Antibacterial tests were conducted via CFU quantification. Furthermore, the bacteria adhesion was determined by the semi-quantitative evaluation of SEM images collected on the coatings’ surface after bacteria incubation. Both the single-doped Ce-HA and co-doped Ce/Si-HA coatings exhibited a better performance with respect to the bare Ti against *P. aeruginosa* and other strains. Ce-HA showed CFU = 8.0 × 10^4^, while Ce/Si-HA showed CFU = 6.2 × 10^4^. Comparing these values with that of pure Ti (8.4 × 10^5^) it was possible to deduce a CFU reduction of 90% for Ce-HA and of 93% for Ce/Si-HA, allowing the conclusion that the co-doped Ce/Si-HA coatings restricted all pathogens better than the single-doped Ce–HA coatings. Bacteria adhesion study revealed a reduced number of bacteria (based on the SEM images) on both the coatings’ surfaces after 24 h of incubation. Moreover, morphological changes occurred on the bacteria grown on the Ce/Si-HA surface, on which broken bacteria walls and shrunken, ruptured cells, with and without interprotoplast leaks, were observed. This finding allowed the conclusion that Ce^4+^ and Si^4+^ ions induced the bacteria’s morphology modification and, in addition, significantly affected the coatings’ characteristics, such as chemical composition, wettability, roughness, etc. These modifications could, in turn, indirectly induce appreciable differences in coating–bacteria interactions.

### 4.2. Staphylococcus Epidermidis

The antibacterial efficacy of Ag-containing HA coatings with 0, 1, and 1.5 wt% of Ag was investigated in [51]. It was tested via the counting of CFU on the coatings’ surfaces. The obtained results showed that bacterial adhesion was significantly reduced on the Ag-HA (1 wt% and 1.5 wt% of Ag) surface (8 × 10^4^ and 25 × 10^4^, respectively) when compared to the Ti and HA surfaces. No significant difference in *Staphylococcus epidermidis* (*S. epidermidis*) adhesion (55 × 10^4^ for both) was observed between the Ti and pure HA surfaces.

Ag-doped HA films with a Ag concentration of 0.5 wt% and 1.5 wt% were tested for antibacterial characteristics in [60]. The antibacterial test modality used in [60] was described in the previous section. The antibacterial performance obtained for the first 24 h was similar to that obtained for *P. Aeruginosa*, where the highest number of dead bacteria (97%) was registered for the coatings with 1.5 wt% of Ag. Nevertheless, for *S. Epidermis*, a significantly different behavior was observed at 24 h and 48 h, when a high percentage of dead cells was registered for both the 0.5 wt% and 1.5 wt% coatings (at 24 h—95% and 97%, at 48 h—57% and 55%, respectively), demonstrating that, for this strain, Ag-HA coatings preserved their antibacterial potential. SEM observations reported that *S. epidermidis* bacteria, unlike *P. Aeruginosa*, developed huge populations on the bare Ti, expanding over the whole surface and forming massive, three-dimensional colonies. Finally, a delamination experiment conducted in tryptic soy broth media for 8 h demonstrated that the Ag release was more effective for the reduction in growth of *S. Epidermis* (1.2 × 10^9^ of viable bacteria) than of *P. aeruginosa* (5.8 × 10^9^).

A Ag-doped CP nano-powder-based ceramic was used to coat Ti pins, and its antibacterial properties were tested in [116]. Antibacterial efficacy against *S. epidermidis* was investigated by CFU counting and presented in CFU/mL. The reported mean values were 1.950 CFU/mL for the Ag-CP-coated group, 6.532 CFU/mL for the undoped coating group, and 8.950 CFU/mL for the uncoated Ti pins. Therefore, the presence of Ag ions significantly reduced bacterial colonization. The minimum inhibitory concentration level for Ag-CP powder was found to be 8 μg/mL. Finally, no presence of free Ag ions was detected in the broth medium after 24 h, suggesting that antibacterial features can be attributed to a “contact killing” mechanism.

An antibacterial assay of Se-doped CP coatings with 0 wt%, 0.6 wt%, and 2.7 wt% of Se concentrations was reported in [117]. CFU counting was used to quantify bacteria activity, while SEM images were collected to observe bacteria adhesion. Furthermore, optical images allowed the evaluation of the biofilm formation after 24 and 72 h. The number of CFU decreased after 12 h of incubation, with a reduction proportional to the Se content in the coating: for 0 wt% Se—3.7 × 10^5^, for 0.6 wt% Se—2.7 × 10^5^ and for 2.7 wt% Se—2.5 × 10^5^. The optical images demonstrated 100% suppression of biofilm formation at the highest Se concentration (2.7 wt%) after 72 h. SEM examination was used to assess *S. epidermidis* adherence. The results showed that the size of bacterial aggregation decreased proportionally to the Se content increase. After 12 h, *S. Epidermidis* adhered to all of the three tested coatings, showing a completely covered surface for the 0 wt% Se coating, reduced bacterial aggregates for the 0.6 wt% Se coating, and isolated bacteria for the 2.7% Se coating. 

### 4.3. Escherichia coli and Staphylococcus aureus 

The antibacterial properties of Ag-HA coatings doped with different concentrations of Ag (0.06 at%; 0.3 at%; 1.2 at%; 4.4 at%; 8.3 at% and 13.7 at%) on Ti were reported in [48]. Antibacterial efficacy was evaluated by counting the CFU of *E. Coli* and *Bacillus subtilis* (*B. subtilis*) strains. All the investigated coatings showed an antibacterial efficacy near to 100%, except for the sample containing 0.06 at% of Ag, which exhibited an antibacterial efficacy of 4%. 

The antibacterial potential of Ag-FHA coatings with different concentrations of Ag ions (0, 5, 10, 20, 40, and 100 ppm) on stainless steels was reported in [56]. The antibacterial activity of the coatings was tested against *S. aureus* and *E. coli* in Mueller–Hinton culture medium via CFU counting. All the Ag-FHA coatings exhibited colony reduction effects equal to or higher than 99.9%, while the absence of a colony reduction effect was detected for FHA. 

The antibacterial characteristics of Zn-CP coatings with low contents of Zn (0.4–0.8 wt%) deposited on Ti-6Al-4V and Ti-6Al-7Nb substrates were described in [73]. Antimicrobial activity was tested against *S. aureus* via the evaluation of CFU area in Petri dishes containing products extracted from Zn-CP and CP coatings. For coatings deposited on Ti-6Al-4V, values of 334 mm^2^ for Zn-CP and 1269 mm^2^ for the pure CP were reported. In the case of samples deposited on Ti-6Al-7Nb, areas of 227 mm^2^ for Zn-CP and 1283 mm^2^ for CP were registered. Therefore, the extracts of Zn-CP were able to diminish CFU areas by 3–4.5 times compared to the pure CP. Based on the obtained results, the authors stated that the low Zn^2+^ concentrations (0.4–0.8 wt%) in the coatings were sufficient to provoke a bacteriostatic effect against planktonic *S. aureus*. Further, an ion release (Ca^2+^, Zn^2+^, PO_4_^3−^) experiment was performed by the immersion of the coatings in a 0.9% NaCl solution to address the Zn bactericidal mechanism. It was observed that, during the first week of release, the low dose Zn-CP (0.4 wt%) coating on Ti-6Al-7Nb was quickly dissolved and the released concentration of Zn increased more than twice compared to the high dose Zn-CaP (0.8 wt%) coating on Ti–6Al–4V. Based on these results, it was concluded that the amount of released Zn^2+^ ions was a more effective parameter for antibacterial activity than the amount of Zn contained in the coatings, and the investigated samples belong to “antibacterial agent release coating” category.

Antibacterial tests on Zn-HA/MWCNT and HA/MWCNT composite films deposited on 316L SS were reported in [70]. They were performed via the evaluation of the inhibition zone against *E. coli*, *S. aureus*, *Shigella flexneri (S. flexneri)*, and *B. subtilis* in Muller–Hinton agar containing broth. The inhibition zone for the Zn-HA/MWCNT composite was 13 mm for *S. aureus* and 17.5 mm for *E. Coli*, while for the HA/MWCNT, values of 8 mm and 12.5 mm for *S. aureus* and *E. coli*, respectively, were registered. Therefore, the inhibition zone around the Zn-HA/MWCNT against Gram-negative *E. coli* was higher than that against Gram-positive *S. aureus*. The authors proposed two possible mechanisms explaining the improved performance of the Zn-HA/MWCNT coating. The first was related to an augmented surface charge, due to the addition of Zn, which helped to adhere a large number of bacteria cells, inducing a direct contact with the Zn embedded on the surface. The second mechanism consisted of the release of Zn ions, able to reduce the bacterial growth in the culture broth.

The antibacterial properties of Mg-HA/MWCNT and HA/MWCNT composite films on 316L SS substrates were investigated in [79]. An antibacterial test was conducted via the evaluation of the width of the inhibition zone, according to a procedure similar to the one described in [70]. The inhibition zone for *S. aureus* was measured to be 11 mm and 8 mm for the Mg-HA/MWCNT and HA/MWCNT films, respectively. For *E. coli* strains, width values of 16 mm and 11 mm for the Mg-HA/MWCNT and HA/MWCNT, respectively, were reported. Therefore, similar to the case described in [70], the widest inhibition zone was registered for Gram-negative *E. coli* with respect to Gram-positive *S. aureus*. Furthermore, in [79], a reduction in the HA crystallite size due to the addition of Mg was reported. This, consequently, resulted in a higher surface area and an increased surface charge, with an improved electrostatic interaction between bacteria and coating. Such modifications in surface properties can induce a better bacteria adherence to the surface and an enhanced interaction between bacteria and the Mg ions embedded in the coatings.

Antibacterial characteristics of Ag-HA/MWCNT and HA/MWCNT nanocomposite films with various concentrations of Ag (1, 3, 5 wt%) on 316L SS substrates were reported in [50]. Antibacterial properties were explored similarly to the procedures described in [70,79]. The results were provided with respect to a positive control—ciprofloxacin. For *S. aureus*, inhibition zone widths of 10, 12, and 13 mm were registered for samples containing 1, 3, and 5 wt% of Ag (30 mm for the positive control), respectively. Under the same conditions, for *E. coli*, values of 16, 17, and 20 mm were obtained for 1, 3, and 5 wt% of Ag, respectively. In this case, the positive control value was 32 mm. Authors suggested that a progressive increase in the Ag content induced the systematic substitution of PO_4_^3−^ groups with AgO_4_^4−^ groups, and this consequently led to a modification of the net surface charge, which, in turn, positively affected the antibacterial efficacy of the Ag-HA/MWCNT coating. Minimum inhibitory concentration (MIC) and minimum bactericidal concentration (MBC) were also determined via broth microdilution against *E. coli*. The obtained values for the composites and positive control were found to be 0.25 mg/mL for 1 and 3 wt% of Ag, 0.125 mg/mL for 5 wt% of Ag, and 0.015 mg/mL for the positive control. The MBC value for 1 and 3 wt% Ag in the composite was detected to be 0.5 mg/mL, while the sample with 5% of Ag showed a value of 0.25 mg/mL. Therefore, the composite coating with an Ag content of 5 wt% was characterized by lower MIC and MBC values with respect to other composites and had the same inhibitory effect with a lower Ag ion concentration in the media solution (0.125 mg/mL for 5 wt% of Ag coatings).

An antibacterial assay was conducted on a Cu-TCP ceramic (0.1 wt% of Cu) prepared via mechano-chemical activation synthesis and described in [78]. The antibacterial activity was tested against *E. coli*, *P. aeruginosa*, and *Salmonella enteritidis* (*S. enteritidis)* by evaluation of the inhibition zone. The Cu-TCP ceramic exhibited specific antibacterial activity against the investigated strains, showing inhibition zones of 0, 22, and 17 mm for *P. aeruginosa*, *S. enteritidis*, and *E. coli*, respectively, while pure TCP ceramic did not possess antibacterial properties. It was concluded that the antibacterial efficacy was related to the presence of the Cu ions in the medium solution. Substitution of Cu in TCP led to a decrease in its crystallinity and an augmented solubility, with a consequent higher release rate of the Cu ions.

**Figure 4:**
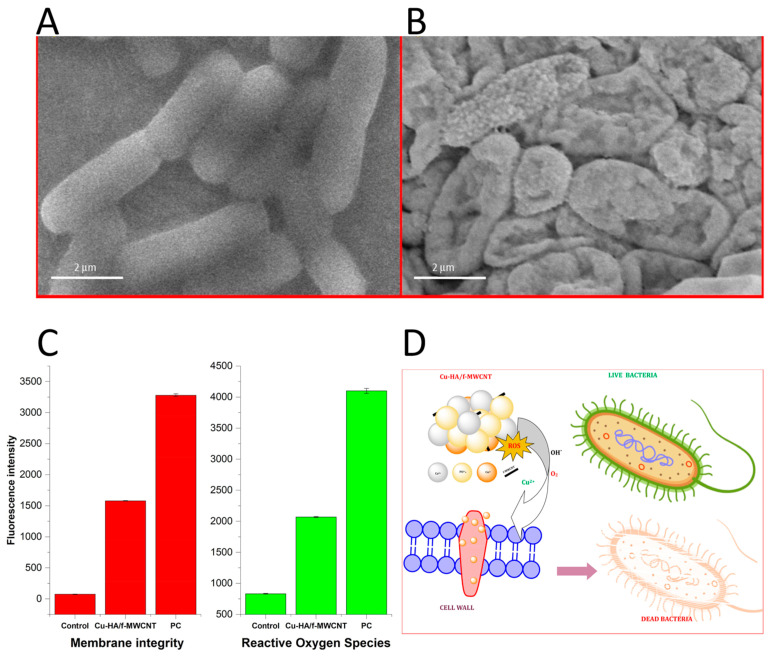
SEM micrographs showing the surface morphology of *E. coli* (**A**) before and (**B**) after Cu-HA/MWCNT treatment. (**C**) Relative fluorescence intensity of MIC of Cu-HA/MWCNT nanocomposite representing the variation membrane integrity of *E. coli* stained with propidium iodide (red bars) and ROS of *E. coli* stained with 2,7-dichlorofuorescin diacetate (green bars). (**D**) Scheme of mechanism of antibacterial activity of Cu-HA/MWCNT nanocomposite. Reprinted with modifications from “International Journal of Pharmaceutics, 590, Sivaraj, D. et al., Tailoring Cu substituted hydroxyapatite/functionalized multiwalled carbon nanotube composite coating on 316L SS implant for enhanced corrosion resistance, antibacterial and bioactive properties, 2020” with permission from Elsevier.

Antibacterial tests on Cu-HA/MWCNT and HA/MWCNT nanocomposite films deposited on 316L SS substrates were carried out in [77]. The applied test procedures were similar to the ones described in [70,79]. Furthermore, cell membrane integrity and intracellular reactive oxygen species (ROS) accumulation assays were conducted by measuring the fluorescence intensity at 617 nm and 535 nm, respectively. Finally, bacteria cell morphology was assessed via SEM investigation after acridine orange/ethidium bromide (AO/EB) dual staining. For *S. aureus*, inhibition zone widths of 8 mm and 13 mm were observed for the HA/MWCNT and Cu-HA/MWCNT, respectively. For *E. coli*, values of 13 mm and 17 mm for the HA/MWCNT and Cu-HA/MWCNT, respectively, were registered. The membrane integrity assay was conducted by measuring the fluorescence radiation from a bacteria suspension in PBS exposed to the MIC of Cu from the Cu-HA/MWCNT coating. The fluorescence intensity was proportional to the amount of disrupted membranes. The obtained results showed that the Cu-HA/MWCNT had a good ability to damage cell membranes, as its intensity (around 1600) was more than 10 times higher than the control H_2_O_2_ (approximately 100), but approximately one half less than the positive control ciprofloxacin (around 3300). The same trend was found for the ROS accumulation assay, where the higher intensity was correlated to a higher presence of the ROS species. These experiments indicated that the Cu ions in the Cu-HA/MWCNT exhibited antibacterial potential through the two main mechanisms: (1) binding of Cu with sulfur–phosphorous-containing compounds present in bacteria’s outer membrane and disruption of the latter, and (2) Cu ions’ ability to diffuse inside the cell, inducing the production of ROS species detrimental for bacteria viability. Finally, SEM analysis confirmed the presence of morphological alterations, such as membrane destruction and the presence of pores in the bacteria walls. The morphology of *E. coli* and schematic representation of antibacterial mechanism are reported in Figure 4.

The antibacterial efficacy of an Ag-TCP coating with 0.08 wt% of Ag on a Ti substrate was tested in [55], according to the modality already described above in Section 4.1. The obtained results indicated that the Ag-TCP film showed an inhibition of approximately 15% for *E. coli* and 17% for *S. aureus*, compared to TCP. Both values were lower compared to that obtained for *P. aeruginosa* (18.5%). The adhesion test revealed a significantly different behavior between *E. coli* and *S. aureus* bacteria, for which adhesion reduction values of 69% and 26%, respectively, were reported. The authors concluded that the results obtained for Gram-negative *E. coli* in the adhesion assay represented the first important step for the inhibition of biofilm formation.

**Figure 5:**
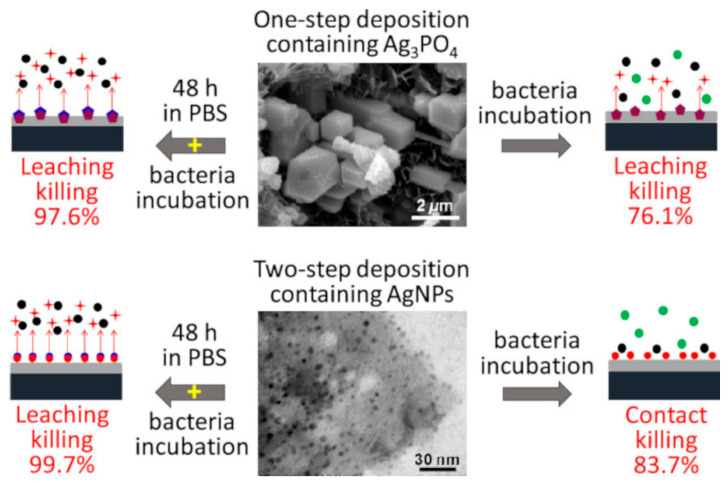
Schematic illustration of the killing mechanisms of Ag-CP coatings. Reproduced from (ACS Appl. Mater. Interfaces 2020, 12, 5, 5531–5541) with permission under CC BY 4.0 license.

Two different Ag-CP coatings were prepared following two different deposition approaches (1—Ag ion introduction and 2—metallic Ag introduction), previously described above in Section 2.1, and their antibacterial behavior was reported in [59]. Three different pre-treatments of samples were performed before the antibacterial tests, namely: immersion in PBS solution, in ultra-pure water, and in culture medium without bacteria, for 48 h. Afterwards, the antimicrobial activity was investigated against Gram-positive *S. aureus* by bacteria reduction evaluation (compared to the undoped CP coating) and by the adhesion test through live/dead staining. The best results for bacteria reduction were achieved for both the Ag-CP(1) (97.6%) and Ag-CP(2) (99.7%), pre-treated in the PBS solutions. The worst performances were obtained for the samples pre-treated in culture medium without bacteria, for which values of 10% and 20% were reported for Ag-CP(1) and Ag-CP(2), respectively. The authors suggested that the diminished antibacterial performance of coatings pre-treated in culture medium can be connected to the sedimentation of proteins, present in the medium and on the coatings’ surface, and their consequent inactivation. More importantly, these findings allowed the authors to conclude that environmental conditions can affect the antibacterial activity more significantly that the coating’s characteristics. Fluorescence micrographs were collected after 6 and 24 h on pure CP, Ag-CP(1) and Ag-CP(2) coatings, on which a portion of the Ti surface was left intentionally uncoated, with the purpose of elucidating antibacterial mechanisms. After 6 h, a few alive and dead bacteria were found on the Ag-CP(1) coating, which indicated a low adhesion rate. Moreover, the uncoated portion of Ag-CP(1) also showed a few alive bacteria cells. Therefore, it can be concluded that, after the first 6 h of incubation, the antibacterial activity was dominated by the leaching killing mechanism through the release of Ag in the culture medium. After 24 h, both alive and dead bacteria were visible on the Ag-CP(1) surface (coated + uncoated), suggesting a decrement of its antibacterial activity. Authors ascribed these results to the interaction of Ag_3_PO_4_, present on the surface of Ag-CP(1), with the bacteria. For the Ag-CP(2), after the first 6 h of incubation, the total number of bacteria (alive + dead) on its surface was higher than on the Ag-CP(1) coating, testifying a higher adhesion. After 24 h, a high number of dead bacteria were detected on the coated portion of the Ag-CP(2) surface, while a considerable number of alive cells were found on the uncoated portion, suggesting that, for Ag-CP(2), antibacterial activity was related to a surface contact mechanism. A schematization of the antibacterial mechanisms of Ag-CP(1) and Ag-CP(2) coatings is reported in Figure 5.

The antibacterial activity of a MgO/Tb,Eu-HA double layer coating on a 316L SS substrate was investigated in [110]. It was determined by measuring the inhibition zone using the disc diffusion method at different concentrations of MgO, Tb, and Eu in the coatings. The width of the inhibition zone showed an increasing trend as the coating material concentration increased, ranging between 8–14 mm for *E. coli* and between 7–12 mm for *S. aureus*, thus demonstrating that the MgO/Tb,Eu-HA coating had higher inhibition activity against Gram-negative *E. coli* than against Gram-positive *S. aureus*. The authors hypothesized that the antibacterial activity was related to the presence of MgO and Tb ions. 

The antibacterial properties of Fe-TCP coatings (with 0, 0.57 and 0.49 at% of Fe) on biodegradable Mg alloys were reported in [118]. The antibacterial effect against *E. coli* and *S. aureus* was evaluated after incubation at 37 °C for 24 h via optical density measurements (600 nm). All tested coating samples significantly inhibited the growth of Gram−negative *E. coli*, showing a decrease of 56% on the coatings’ surface compared to the control sample (100%). The average growth rate of Gram-positive *S. aureus* was not less than 94%, compared to the control. No significant differences in antibacterial properties were observed between the Fe-TCP and TCP coatings with respect to both the bacteria strains. According to the results obtained in [118], the coatings’ antibacterial activity was not connected to the presence of the Fe ions.

An antimicrobial assay was performed for a Mn,Sr-TCP coating on a ZnLi alloy in [94]. Antimicrobial testing was assessed against *S. aureus*, *E. faecalis*, *Salmonella typhimurium (S. typhimurium)*, and *E. coli* bacteria strains and *C. albicans* fungus by determination of the growth rate by optical density (600 nm) measurements. The inhibition of growth was registered for all the investigated strains and fungus: 9% for *E. coli*, 13% for *S. aureus*, 10% for *P. aeruginosa*, 10% for *E. faecalis*, and 44% for *C. albicans*, the latter exhibiting the best result.

A Sr,Zn-HA coating with 5 wt% of Sr and Zn ions was deposited on Ti and subsequently underwent heat treatment at 500 °C and 600 °C in [119]. Antibacterial efficacy against colonization and adhesion of *E. coli* was measured by the evaluation of fluorescence imaging after live/dead staining treatment. The Sr,Zn-substituted HA coating showed a higher antibacterial efficacy than the non-substituted HA one. Indeed, only a few alive bacteria were found on the surface of the Sr,Zn-HA coating. Moreover, the increase in temperature of the heat treatment led to the enhancement of the antibacterial efficacy, confirmed by the higher number of dead bacteria. The authors [119] also carried out the Zn^2+^ release experiment, which revealed a higher release of Zn ions after the heat treatment. A higher release of Zn^2+^ was correlated with the augmented antibacterial properties. It should be noted that the presence of Sr did not influence the antibacterial effect of Zn, likely correlated to the ability of Zn to form strong bonds with the bacterial membrane, causing structural changes and impaired mobility and proliferation.

Antibacterial investigation of HA and co-substituted Sr,Zn-HA coatings (5%Sr,10%Zn-HA, 10%Sr,5%Zn-HA, and 10%Sr,10%Zn-HA) on Ti was described in [90]. The antibacterial ability of co-substituted HA coatings was tested against *E. coli* using live/dead fluorescence staining. The higher concentrations of Zn^2+^ and Sr^2+^ resulted in better antibacterial performance. The best results were obtained for the 10%Sr,10%Zn-HA coating, with very few alive bacteria found on its surface. It was noted that the antibacterial properties of Zn^2+^ ions were related to their ability to damage cell membranes and to hinder enzymes’ activity. Furthermore, co-substitution with Sr was reported to induce a synergistic effect on the incremented antibacterial activity.

A La/Tb-HA/CS-CA/TN multi-layer coating with a La concentration of 0.05M and Tb concentration of 0.075 M and its antibacterial characteristics were described in [105]. The antibacterial activity of this coating was tested against *S. aureus* and *E. coli* by measuring the inhibition zone at four different concentrations of the material (25, 50, 75, and 100 μL) from the scraped coating sample. Extension of the inhibition zone followed a constant increasing trend as the material concentration increased. The width of the inhibition zone was in the range of 11–22 mm for *S. aureus* and of 20–27 mm for *E. coli*. Different results obtained for Gram-positive *S. aureus* and Gram-negative *E. coli* were attributed to the different structure of the cell walls of these strains. 

The antibacterial efficacy of Ag-HA, Sr-HA and Ag,Sr-HA powder samples with Ag^+^ and Sr^2+^ ions at different concentrations (0, 1, 2.5, and 5 mol%) for each ion species was reported in [87]. Antibacterial activity was tested against *E. coli* and *S. aureus* by evaluation of the inhibition zone. In addition, the surface morphology, after diffusion assay, was investigated via SEM. The Ag-HA powder sample showed an evident antibacterial activity, while that of the Sr-HA did not, as was observed by the absence of the characteristic halo around the sample. The antibacterial activity of the Ag-HA and Ag,Sr-HA samples did not increase linearly with the increase in the Ag^+^ ions concentration. Moreover, it was observed that the inhibition zone for *S. aureus* bacteria was divided into two areas. A fuzzy edge, observed on the border of the inhibition zone, indicated some degree of resistance of Gram-positive bacteria to the Ag^+^ ions. The thickness of the peptidoglycan layer in Gram-positive bacteria may protect them from the influx of Ag^+^ ions, which could explain the observed resistance. SEM microscopy showed that the Ag,Sr-HA sample had a synergistic effect on the inhibition of the bacteria attachment, with no viable *E. coli* and *S. aureus* cells detected on the surface. Ag-HA samples also inhibited the bacteria attachment, damaging the cytoplasmic membrane. Finally, the Sr-HA and Ag,Sr-HA samples showed the presence of smaller *S. aureus* colonies compared to *E. coli*, suggesting a higher antimicrobial potential of Sr against the *S. aureus* strain.

The antibacterial activity of Ag,Sr-FHA, Ag-FHA, and pure FHA coatings on Ti was studied in [120]. Antibacterial effect against *E. coli* and *S. aureus* was first evaluated by observation of the inhibition zone. Afterwards, the bacteriostatic rate test was assessed via measuring the optical density of bacteria suspension and, finally, a live/dead bacteria experiment was conducted via confocal microscopy observations after a bacteria staining procedure, in which each sample was covered with SYTO-9 dye for live bacteria and propidium iodide (PI) dye for dead bacteria and kept in the dark for 15 min. Qualitative evaluation of the inhibition zone allowed the authors to conclude that the coatings containing Ag exhibited antibacterial properties, as was observed from the presence of a characteristic halo surrounding these coatings. No presence of the halo was observed for the pure FHA coating. The bacteriostatic test revealed that, for *E. coli*, sterilization rates of 95.8% and 96.2% were calculated for Ag-FHA and Ag,Sr-FHA coatings, respectively. Very similar values (95.9% for Ag-FHA and 96.3% for Ag,Sr-FHA) were found for *S. aureus*. These findings confirmed the antibacterial effect of Ag for both the bacteria strains. Finally, the live/dead assay showed a considerable number of alive bacteria with undamaged membrane on the FHA coating, whereas, for the Ag containing coatings, a significant increase in dead bacteria was registered, confirming the high antibacterial potential of Ag.

Zn and Mg-substituted CP coatings with different ratio of Mg and Zn were deposited on Ti, according to the modality described in Section 2.1.4, and their antibacterial characteristics were reported in [93]. The antibacterial activity of the coatings was investigated using the diffusion disk technique against Gram-negative *E. coli*. The inhibition zones were measured for each coating: 39 mm for 20Zn–DCPD, 31 mm for 10Mg-14Zn-DCPD, 25 mm for 20Mg-7Zn-DCPD, and 0 mm for 30Mg-DCPD. The increase in the Zn content in the coatings led to the enhancement of the antibacterial properties, with the largest inhibition zone observed for the sample with 20 mM of Zn (20Zn-DCPD). Authors concluded that Zn ions can prevent bacterial growth by disrupting DNA replication and metabolic enzymes, as well as by damaging cell membranes through the production of ROS.

The antibacterial activity of the HNT-CS-MHA-Ti (M = Zn and Mg) composite coating on Ti was evaluated in [102] (Section 3.2). It was tested by measuring the inhibition zone of bacteria, cultured with various quantities of the material from the scraped coating, namely 20, 40, 65, and 80 μL. Incremental amounts led to a constant increase in the inhibition zone span, from 3 to 13 mm for *E. coli* and from 1 to 10 mm for *S. aureus*. The authors hypothesized that CS and Zn ions can have a key role in the process of killing bacteria, due to the ability of CS to chelate trace ions, such as Zn, which can hinder the enzymatic activity of bacteria.

The antibacterial efficacy of the Ce-HA/collagen (0.48 at% of Ce) and HA/collagen coatings on Ti was investigated in [104]. Antibacterial effects against *E. coli* and *S. aureus* were assessed by calculating the bactericidal ratio (BR%) from the number of CFU formed on the Ce-HA/collagen and HA/collagen coatings. The Ce-HA/collagen coating was able to reduce up to 92% of *E. coli* and up to 73% of *S. aureus*, after 24 h of incubation. In comparison, only 2% of *E. coli* and 4% of *S. aureus* were eliminated by the HA/collagen coating. The authors [104] also reported a decreased crystallinity of the HA matrix upon Ce addition. This can induce an increased solubility with a consequent higher release of Ce^4+^ ions in the solution, which might be correlated with an improved antibacterial efficacy. Furthermore, Ce^4+^ ions could create a positive charge on the coating’s surface, which may result in an improved electrostatic attraction between bacteria and the apatite layer, enhancing the bacteria–ion interactions. 

Zn-HA, Cu-HA (with 2.5 at% of both Zn and Cu ions), and pure HA coatings on Ti were tested for antibacterial characteristics in [121]. Antibacterial activity was assessed against *E. coli* and *S. aureus* by collecting SEM and epifluorescence DAPI micrographs. The collected images were used to evaluate the degree of colonization of the coatings’ surfaces and the bacteria’s adhesion. Furthermore, SEM and DAPI images were used to estimate the CFU number by visual inspection. It was observed that both the Zn-HA and Cu-HA coatings (very similar results were obtained for both) inhibited *S. aureus* growth by up to 75%. The results reported for *E. coli* showed that the Cu-HA coating had an inhibitory effect of 42%, while that of the Zn-HA was 14%. A qualitative evaluation of SEM and DAPI images showed that, for *E. coli*, the adherence remained unaltered for all the investigated coatings. Nevertheless, a decrease in the biofilm formation was observed for *E. coli* colonies grown on the Zn-HA and Cu-HA coatings. For *S. aureus*, in addition to a reduced biofilm formation, the adherence was also reduced by one order of magnitude compared to the pure HA. The authors [121] concluded that, even though both bacteria strains were sensitive to Zn and Cu ions, the best results were obtained for *S. aureus*, in terms of inhibition of growth and of adhesion reduction.

The antibacterial properties of single-doped Ag-HA/TiO_2_ and multi-doped Sr,Ag-HA/TiO_2_ and Si,Sr,Ag-HA/TiO_2_ composite coatings on Ti were investigated in [96]. Antibacterial testing against *S. aureus* was conducted via the analysis of bacteria inhibition after an incubation time of 36 h. A growth suppression rate of 100%, 99%, and 99.5% was reported for Ag-HA/TiO_2_, Sr,Ag-HA/TiO_2_, and Si,Sr,Ag-HA, respectively. The antibacterial activity was ascribed to the Ag ions in the coatings, which were released from the host HA matrix and, therefore, acted as free ions in the solution. Indeed, the Ag release experiments performed for the Sr,Si,Ag-HA/TiO_2_ coating after 14 days of immersion in PBS revealed a cumulative concentration of Ag^+^ of 0.711 mg L^−1^ in the solution.

The antibacterial properties of four types of coatings, such as Ca_10_(PO_4_)_6_(OH)_2_, Ca_9_._95_Ag_0_._1_(PO_4_)_6_(OH)_2_, Ca_9_Sr(PO_4_)_6_(OH)_2_, and Ca_8_._95_SrAg_0_._1_(PO_4_)_6_(OH)_2_, (labelled as HA, Ag0.1, 10Sr, and Sr/Ag, respectively) on Ti were described in [85]. Antibacterial tests were carried out via evaluation of the inhibition zone and by bacteria number counting of *S. aureus* and *E. coli*. Bacteria morphology was investigated by SEM and TEM microscopies. After 24 h of incubation at 37 °C, no inhibition zone was observed on pure Ti, HA, and 10Sr for both *E. coli* and S. *aureus* strains. Instead, all the Ag-containing samples displayed well-observed inhibition zones. The widths of the inhibition zones against *S. aureus* (11.0 mm and 10.5 mm for Ag0.1 and Sr/Ag, respectively) were smaller than those observed against *E. coli* (12.5 and 11.5 mm for Ag0.1 and Sr/Ag, respectively). It is worth noting that both the Ag0.1 and Sr/Ag coatings contained the same quantity of Ag. It was revealed that, after 24 h of incubation, *E. coli* was completely eliminated (100%) by Ag 0.1 and almost completely eliminated (99%) by Sr/Ag. For *S. aureus*, a decrease of 96% and 95% was registered for Ag0.1 and Sr/Ag, respectively. These results were in accordance with the outcomes obtained by the inhibition zone measurements, confirming a slight decrease in the antibacterial potential of coatings upon the addition of Sr. For both the bacteria strains grown on the Ag0.1 and Sr/Ag coatings, a significant decrease in density was observed in the SEM images. SEM and TEM micrographs highlighted morphological alterations for both strains, such as ruptured membranes and separated peptidoglycan layers.

The antibacterial potential of single-substituted Zn-FHA, Cu-FHA, and Ag-FHA and co-substituted Zn,Cu-FHA coatings on 316L SS substrate was studied in [122]. All the ion precursors were added at a concentration of 100 ppm for each ion species. Antibacterial testing against *S. aureus* was conducted via CFU counting and reported as percentage reduction in CFU, compared to control (a saline solution), after 48 h of incubation. All the ion-substituted coatings presented an almost complete reduction (higher than 99.99%) in active bacteria. In contrast, for FHA, a value of 0% was registered. The inclusion of Zn, Cu, and Ag ions led to a decrease in the HA crystallinity with a consequent increase in the dissolution rate, allowing metal ions to diffuse in culture medium, thus improving the antibacterial performance.

## 5. Perspectives

The research of alternative non-antibiotic-based solutions to fight bacterial infections and antibiotic resistance rely on the comprehension of mechanisms underlying the interactions between bacteria and antibacterial systems. In particular, in this review, we analyzed research articles dedicated to CP-based coatings containing doping ions, developed with the aim of providing an effective, suitable and tailored solution as a valid support/alternative to antibiotics therapy. Coatings were mainly characterized by high surface area and low thickness. It was crucial to analyze all the chemical and physical-chemical properties related to the surface of the coating systems and the way they interact with bacteria. 

During the literature search performed for this review, it became more and more clear that in almost none of the reported articles were the investigation of superficial charge and zeta potential (ZP), their correlation with bacteria-coating interaction, and possible practical implications taken into account. This evidenced a lack of a more complete and deep knowledge of bacteria–coating interactions, with consequent theoretical and practical ramifications. It is worth noting that, in general, during the last few years an increasing number of scientific works in the field of materials science have focused attention on the investigation of the superficial charge and ZP, but such studies are scarce for CP coating systems [87,123,124,125]. Despite the fact that the measurement of ZP for a complex, three-dimensional, living and evolving system, such as a bacterium, represents a challenge, continuous efforts in this direction provided a reliable method for its quantification [126]. Several works presented evidence of the correlation between ZP, measured at the bacteria surface, and the nature of different strains (in particular, differences between Gram-positive and Gram-negative bacteria) [127], their degree of maturation, eventual mutated sub-populations, and their response to environmental conditions, such as pH, temperature, presence of specific electrolyte in solutions, etc. [128,129,130]. Induced variations in the ZP of bacteria surfaces imply significant modifications in the bacterial biology (membrane permeability, metabolism, protein production, etc.) [131]. These modifications should represent a starting point for the design of new antibacterial systems (such as, for example, metal ion CP-based coatings), taking into consideration the surface charge and its key role for a fine-tuned targeting of the bacteria surface [132,133]. To sum up, major attention should be given to the deepening of knowledge on surface charge and its implications in bacteria–coating interactions, as it may represent an important tile in the development of the effective antibacterial solution mosaic.

Currently, a number of studies are devoted to the substitution of Ca^2+^ positions in the CP host matrix by mono-, di-, and trivalent ions, with scope to create new bioactive materials with outstanding functional properties [114,134,135,136]. Among them, multi-substitution strategy is expected to provide the combined advantages offered by the presence of two (or more) different ions in the host CP structure. However, in this case, the structural characteristics of a particular CP (HA or β-TCP or other) should be taken into consideration. For example, in the case of co-substitution in β-TCP with small divalent ions, such as Zn^2+^, Mg^2+^, Cu^2+^, etc., these ions compete for the octahedral M5 site occupation in the β-TCP crystal lattice, and the excess concentration of substitution ions leads to the formation of impurity phases, which may cause opposite and undesired effects. The limits of the single-phase solid solutions were found for double substitutions in the β-TCP structure and described in [137]. Interestingly, in the same work, it was also discovered that the incorporation of Sr^2+^ ions along with the other divalent metal ions allows the extension of the limit of solid solutions due to the unit cell enlargement. Moreover, it was shown that active dopant ions should be located in different crystal sites to maximize the antimicrobial effect.

It should be added that the biological performance of the dopant ions may depend on their occupied position in the crystal lattice and on their concentration. For Cu^2+^ substitution in β-TCP, the concentration-dependent behavior of the inhibition of bacteria growth was observed in [138]. Furthermore, the antimicrobial properties were found to depend on the ion release behavior, which, in turn, was dependent on the Cu^2+^ position in the β-TCP crystal structure: Cu^2+^ location in the large M3 polyhedrals led to a noticeable increase in the ion release. Therefore, further studies of the site-selective arrangement of bioactive ions should be performed in order to enhance the antimicrobial potential of doping ions.

**Figure 6:**
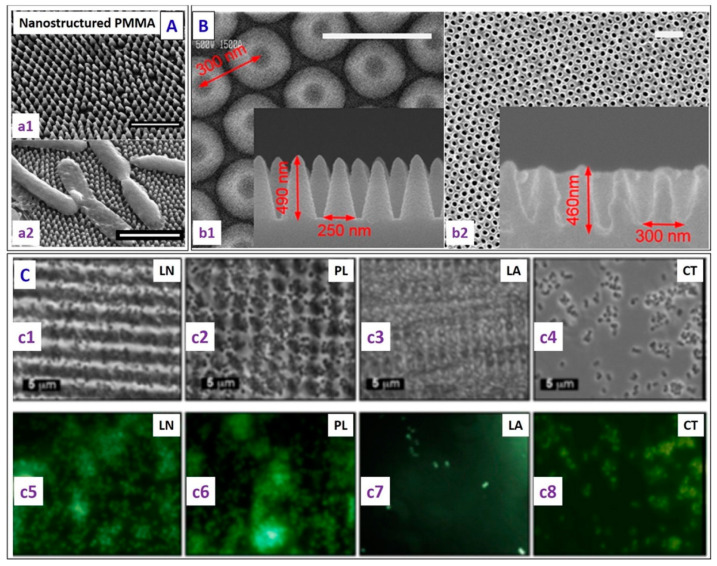
(**A**) (a1) Representative SEM image of PMMA surfaces at a 30° tilt, where a two-step lithography process was carried out to replicate the nanostructures on the surface of the cicada wing in PMMA (scale bar 1 μm). (a2) Representative SEM micrograph of *E. coli* on patterned PMMA surfaces (scale bar—2 μm), showing deflated bacteria draped across several PMMA pillars (whereas they retained the rod-shape on the control flat PMMA surface). (**B**) SEM images of the nanostructured surface: (b1) top view and cross-sectional view (inset) of the silicon master surface, and (b2) nanostructured pattern on the PMMA film and a magnified cross-sectional image (inset) (scale bar—500 nm). (**C**) (c1) Attachment of *S. aureus* to patterned PS wafers (c1) LN—line structure, (c2) PL—pillar structure, (c3) LA—lamella structure and (c4) CT—control surface. Corresponding fluorescence microscopic images (**bottom**) showing more *S. aureus* (green spots showing the number of bacteria present) adhesion on the (c5) line (LN), (c6) pillar (PL) and (c8) control surfaces than the (c7) lamella (LA) structures. [reproduced with permission from Ref. [139] permission under CC BY 4.0 license].

The ion-doped CP-based coatings described in this review represent chemical methods of surface modifications with scope to impart antibacterial properties. In order to prolong and enhance the antibacterial effect, a synergy with other possible suitable approaches should be considered. Among them, the antimicrobial effect of surface topography is very promising. Indeed, surface nano-patterning is a rapidly growing area of research. The design of high aspect ratio nano-patterns providing a bacteria repellent surface and preventing biofilm formation is rather challenging. Representation of the nano-patterned surfaces of PMMA (poly(methyl methacrylate)) is given in Figure 6. 

The analysis of the literature reveals that design parameters, such as optimum size, shape, and density of nano-patterns for maximizing bactericidal behavior, are not yet well defined. An overview of antibacterial behavior of naturally occurring and bio-inspired nanostructured surfaces is presented in [139], whereas upcoming technologies applying cell–surface interactions at the nano-scale and the influence of nano-features’ size, shape, and spacing on cell response are described in [140]. 

Another possible approach that can be combined with ion-doped CP-based coatings deposition is related to antimicrobial peptides, which have recently received great interest as potential next-generation natural antibiotics [141]. Such peptides are small bioactive proteins, produced by living organisms and characterized by a broad-spectrum activity against numerous pathogenic micro-organisms. The interest in antimicrobial peptides further increased during the Severe Acute Respiratory Syndrome Coronavirus 2 (SARS-CoV-2) pandemic as new antiviral molecules to counteract COVID-19 [142]. 

## 6. Conclusions

Ion substitutions in CP-based coatings were shown to have strong influence on the coatings’ properties. Firstly, the introduction of one or more metal ions induced chemical and physico-chemical modifications at the micro- and/or nano- scale of the host CP matrix, such as lattice parameters, crystallinity degree, local charge, formation of weak bonds (i.e., Van der Waals or hydrogen interactions), etc. These modifications are mainly correlated with the chemical nature of the ion, its concentration, the ionic radius of the introduced ion species, and its charge (oxidation state). The micro-scale modifications directly influence the macro-scale properties, such as stability of the CP matrix, its solubility, wettability, mechanical properties, degradation and corrosion behavior, and its bioactivity and cytocompatibility. When co-substitution with different metal ion species was investigated, it was observed that the co-presence of various ions can lead to different results. In some cases, a synergistic effect was reported; in other cases, the reported results demonstrated that co-ions could act in opposite directions, imparting contrasting characteristics. However, the majority of the reviewed works were focused on the behavior of individual ions, without taking into account mutual ion interactions. Composite coatings composed of compounds of different natures demonstrated the possibility of inducing significant changes in the CP coating properties, according to the nature of the additional compound(s) and its (their) relative quantity in the composite. Different types of additional compounds were utilized, such as polymers, biopolymers, inorganics, and different types of nanotubes. In the reviewed literature, the reported composite coatings were a mix of the CP phase and additional compound, or a multilayer structure. The presence of additional components proved to affect and, in particular, to enhance several properties of the coatings, at the macro- and micro-scales. Indeed, it was observed that polymers and biopolymer species were able to form molecular bridges (strong bonds) between (bio)polymers and the CP particles at the micro-scale, which strongly influenced the coating properties, such as wettability, adhesion, bioactivity, and biocompatibility. Inorganic and nanotube-shaped species, often used in a multi-layer configuration, were able to influence coatings’ degradation and to improve the corrosion behavior, since they acted as protective layers against corrosive solutions. Therefore, it was demonstrated that the coating properties can be improved by the additional components, with a possibility to tune specific characteristics. Finally, it is important to note that the coatings described in the present review were shown to possess antibacterial properties, not only due to the direct or indirect action of metallic ions, but also due to the influence of other coatings’ features. It was observed that coatings can exhibit antibacterial potential through two main mechanisms: contact killing and ion release. The contact killing mechanism is related to the ability to modulate the adhesion of bacteria to the coating surface, providing a close contact with the species present on the surface. Adhesion of bacteria is mainly related to wettability, surface morphology (roughness), and surface charge. Moreover, the enhanced adhesion of bacteria to the surface can hinder its mobility and induce membrane rupture. The second mechanism is related to the release of antibacterial ions in a solution through their diffusion from the surface. This characteristic is regulated by the stability of the coatings, their solubility, adhesion to the substrate (detached pieces of coating can pass into solution), and anti-corrosion properties. Once in contact with bacteria, metal ions can express their antibacterial potential mainly by three mechanisms: enzymatic inhibition, oxidative stress, and membrane rupture. Various literature references reported different methods to assess antibacterial efficacy, to elucidate coating mechanisms and ion–bacteria interactions, and to clarify mechanisms of fighting against bacteria growth and proliferation. In conclusion, the incorporation of various ions into the CP structure was shown to enhance the biological and antibacterial properties of the reported coatings. Finally, ion-doped CP-based coatings offer great potential for the development of advanced biomaterials for various biomedical applications. Further research is needed to optimize the properties of these coatings and improve their clinical potential.

## Data Availability

Not available.
